# Supplementary figures and images for: Communication between the stem cell niche and an adjacent differentiation niche through miRNA and EGFR signaling orchestrates exit from the stem cell state in the Drosophila ovary
Source: PLoS Biol. 2024 Mar 21;22(3):e3002515. doi: 10.1371/journal.pbio.3002515 (PMC10986965; doi:10.1371/journal.pbio.3002515)

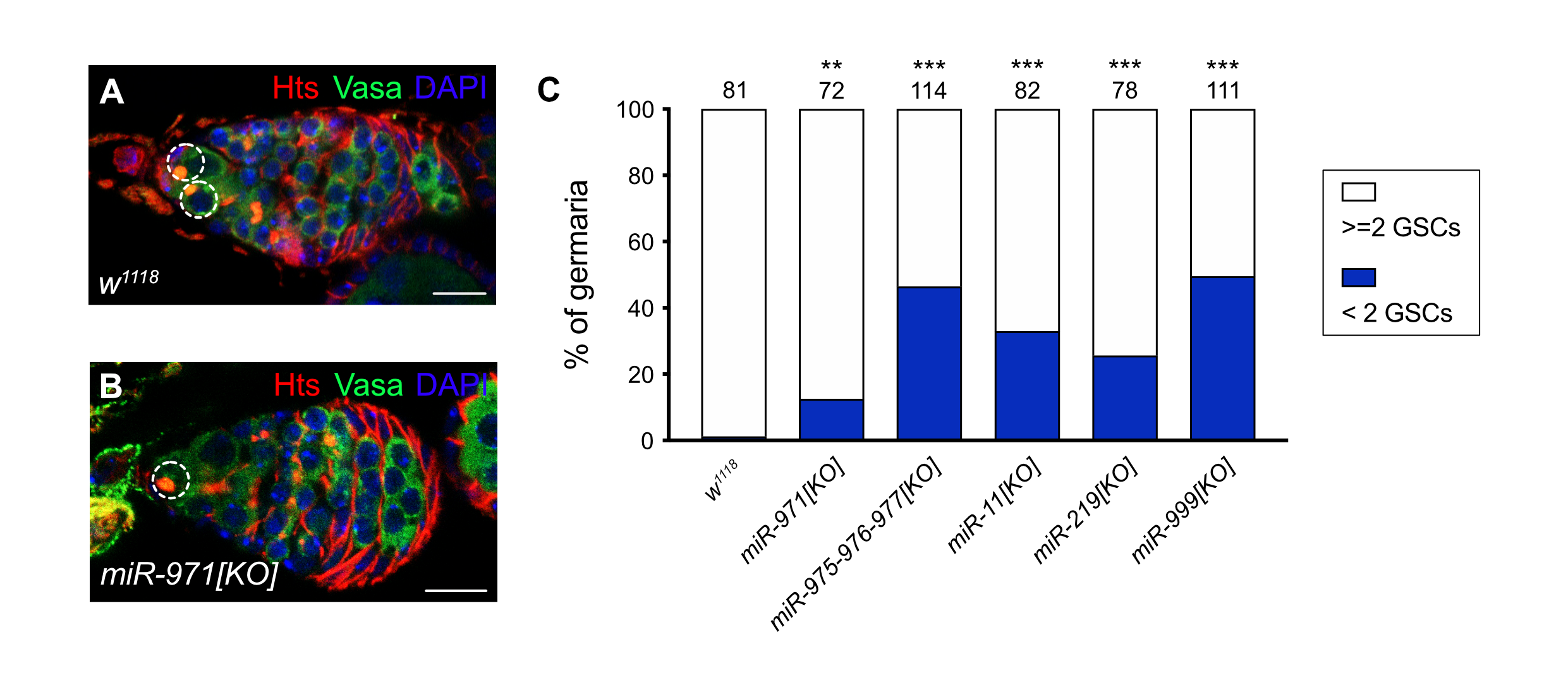

Supplement: S1 Fig — (A and B) Germaria from wild-type (A) or a miRNA mutant (B) were stained with antibodies to Hts (red) and Vasa (green), together with DAPI (blue), to label spectrosomes or fusomes, germline cells, and nuclei, respectively. Scale bar: 10 μm. (A) The control germarium contained 2 GSCs (indicated by dashed circles). (B) Germarium of the miR-971[KO] mutant contained only 1 GSC (indicated by dashed circles). (C) Percentage of germaria with fewer than 2 GSCs in wild-type (w1118) and the designated homozygous miRNA mutants. The number of analyzed germaria is shown above each bar. Significance was determined by Fisher’s exact two-sided test (** P < 0.01; *** P < 0.001). The raw data underlying panel C are available in S1 Data. (TIFF) [file pbio.3002515.s001.tiff]

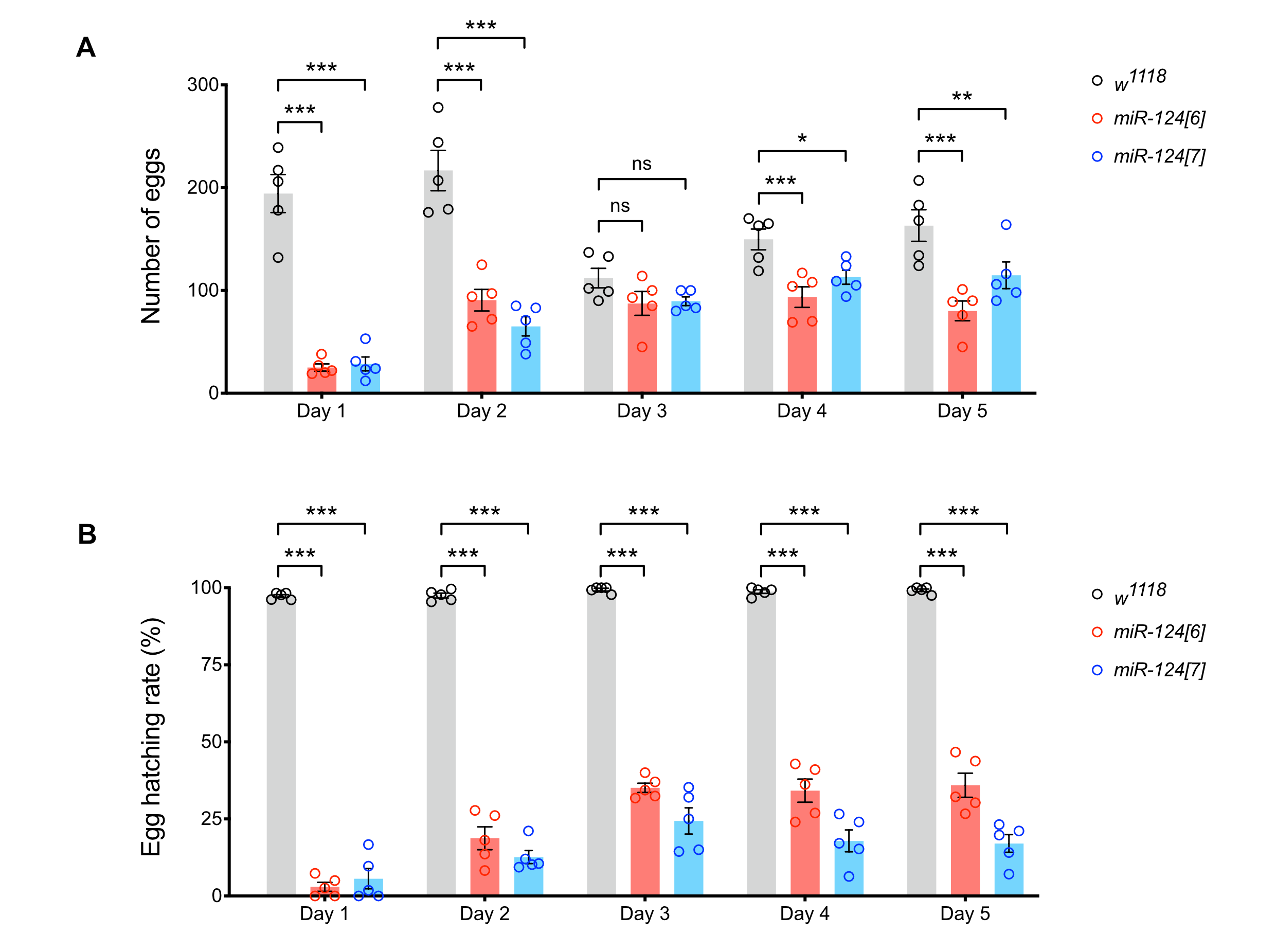

Supplement: S2 Fig — (A) The daily number of eggs laid by the w1118 control (gray) and the miR-124[6] (red) or miR-124[7] (blue) mutants. (B) Hatching rate of the oviposited fly eggs in (A). Five biological replicates were performed. Data represent the mean ± SEM. Significance was determined by two-way ANOVA with Dunnett’s multiple comparisons test (* P < 0.05; ** P < 0.01; *** P < 0.001; ns: not significant). The raw data are available in S1 Data. (TIFF) [file pbio.3002515.s002.tiff]

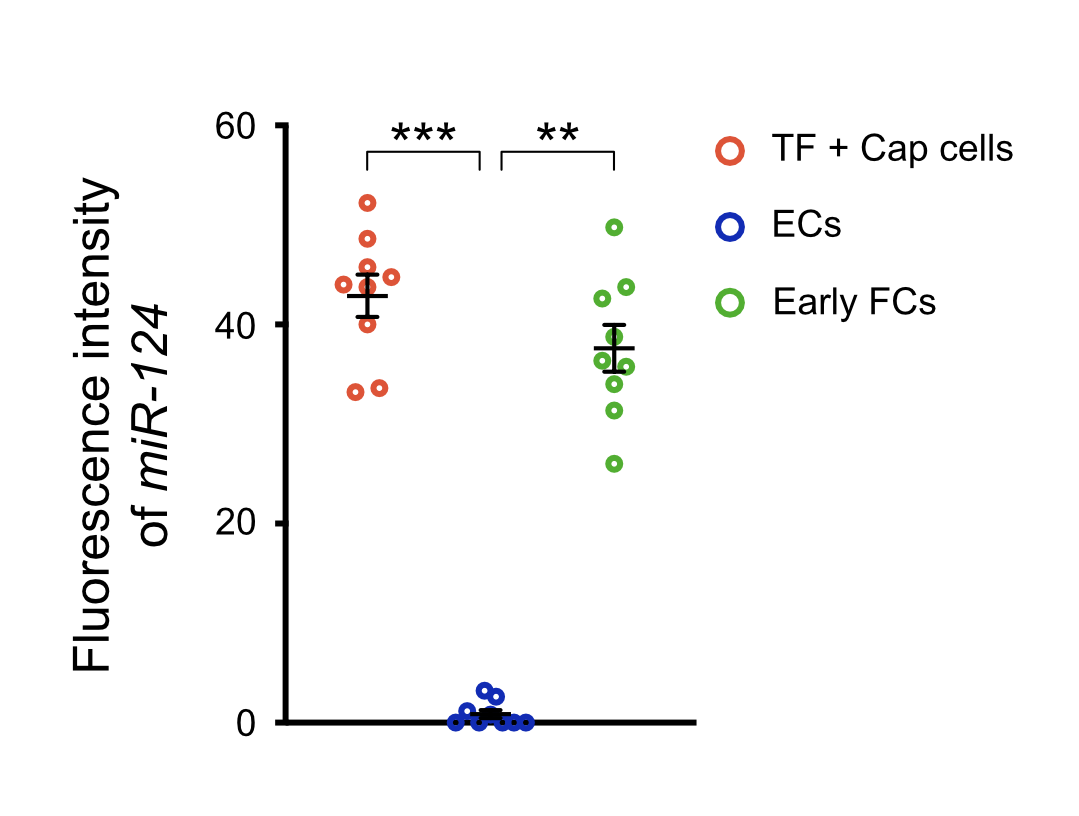

Supplement: S3 Fig — Quantification results on miR-124 expression in TF and Cap cells (red circles), ECs (blue circles), and early FCs (green circles) per germarium. Each plot indicates the mean intensity from 5 cells for each germarium (n = 9 Drosophila germaria were examined for each group). Data are presented as the mean ± SEM. Significance was determined by Kruskal–Wallis one-way ANOVA with Dunn’s test (** P < 0.01; *** P < 0.001). The raw data are available in S1 Data. (TIFF) [file pbio.3002515.s003.tiff]

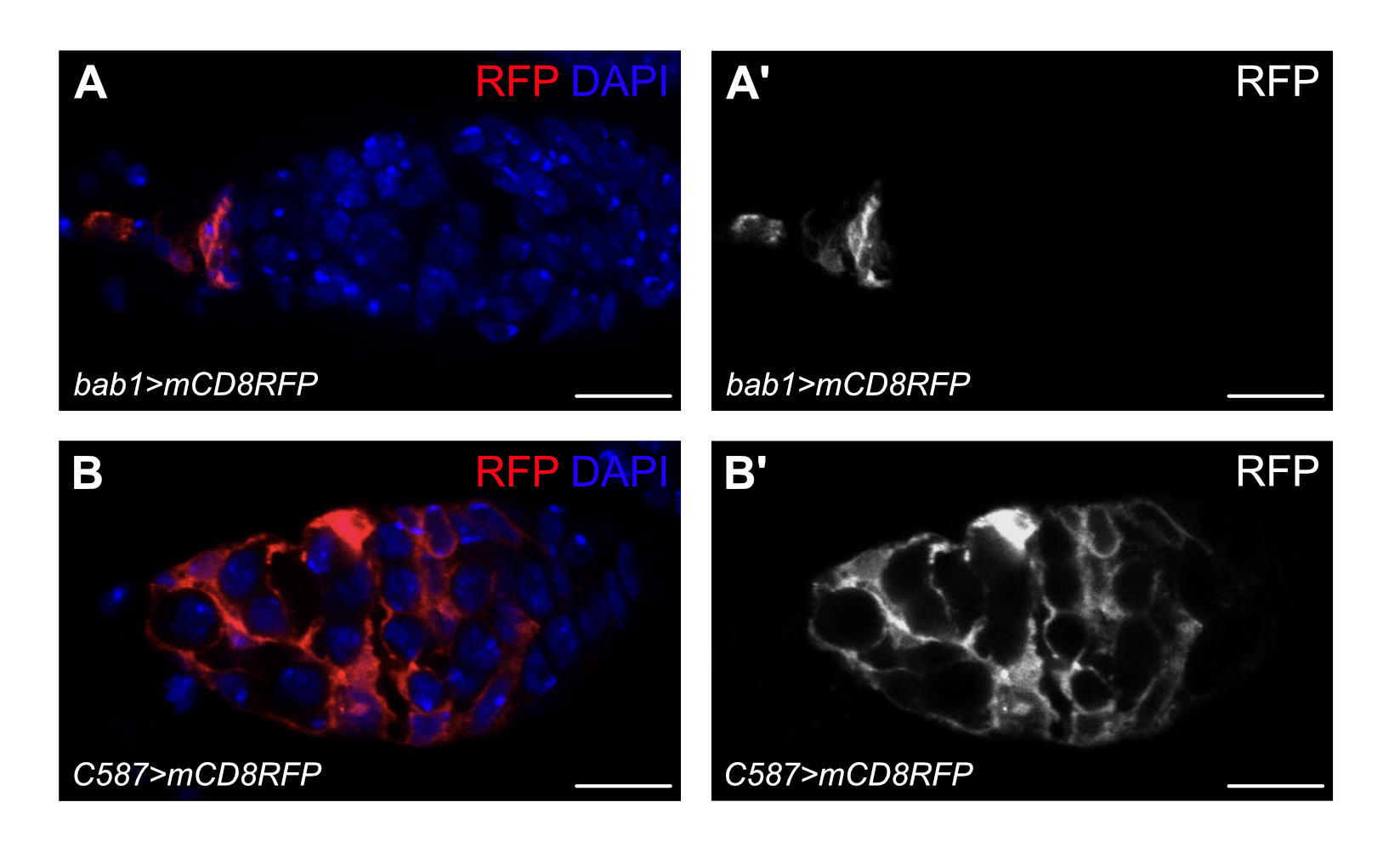

Supplement: S4 Fig — (A–A’) UAS-mCD8RFP and bab1-GAL4 were used to indicate the bab1-GAL4 expression pattern (red) in the germaria, which indicated bab1-GAL4 is specifically expressed in TF and Cap cells but not ECs. (B–B’) UAS-mCD8RFP and C587-GAL4 were used to indicate the C587-GAL4 expression pattern (red) in the germaria, which indicated C587-GAL4 is specifically expressed in ECs but not TF or Cap cells. (A) and (B) show the merging of the 2 channels of RFP and DAPI (blue); (A’) and (B’) show RFP-stained images in black and white. Scale bar: 10 μm. (TIFF) [file pbio.3002515.s004.tiff]

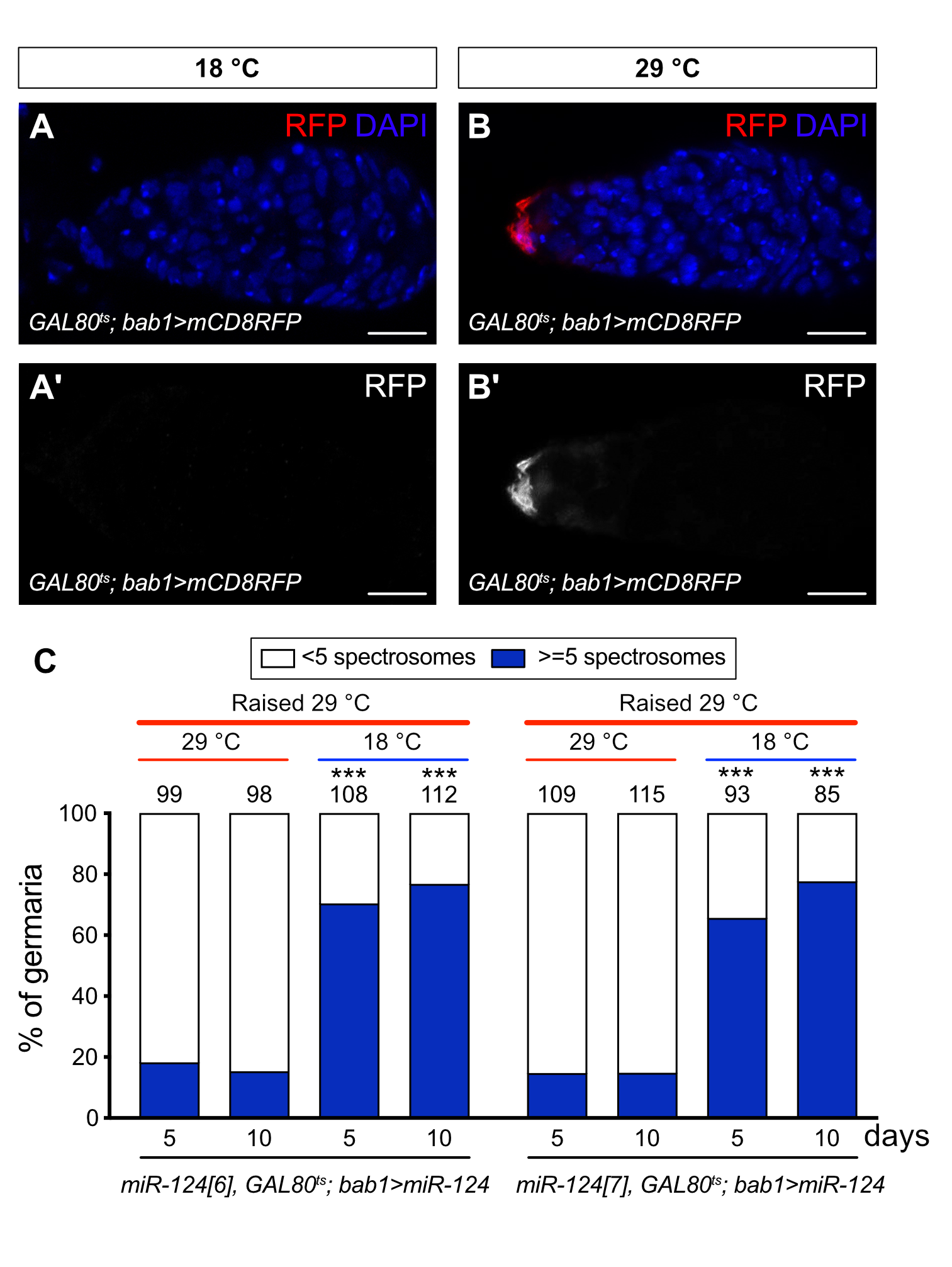

Supplement: S5 Fig — (A–B’) Animals carrying UAS-mCD8RFP and GAL80ts; bab1-GAL4 were raised at 18°C up to eclosion and then maintained at 18°C (A–A’) or 29°C (B–B’) for 5 days before ovary dissection. GAL4 activation of UAS-mCD8RFP was suppressed at 18°C while was activated at 29°C. (A) and (B) show the merging of the 2 channels of RFP and DAPI (blue); (A’) and (B’) show RFP stained images in black and white. Scale bar: 10 μm. (C) Animals carrying UAS-miR-124 and GAL80ts; bab1-GAL4 were raised at 29°C up to eclosion and then maintained at 29°C or 18°C for the number of days indicated before ovary dissection. The percentage of germaria carrying 5 or more spectrosome-containing cells is shown, and the number of analyzed germaria is above each bar. Significance of 29°C vs. 18°C for the same time period was determined by Fisher’s exact two-sided test (*** P < 0.001). The raw data underlying panel C are available in S1 Data. (TIFF) [file pbio.3002515.s005.tiff]

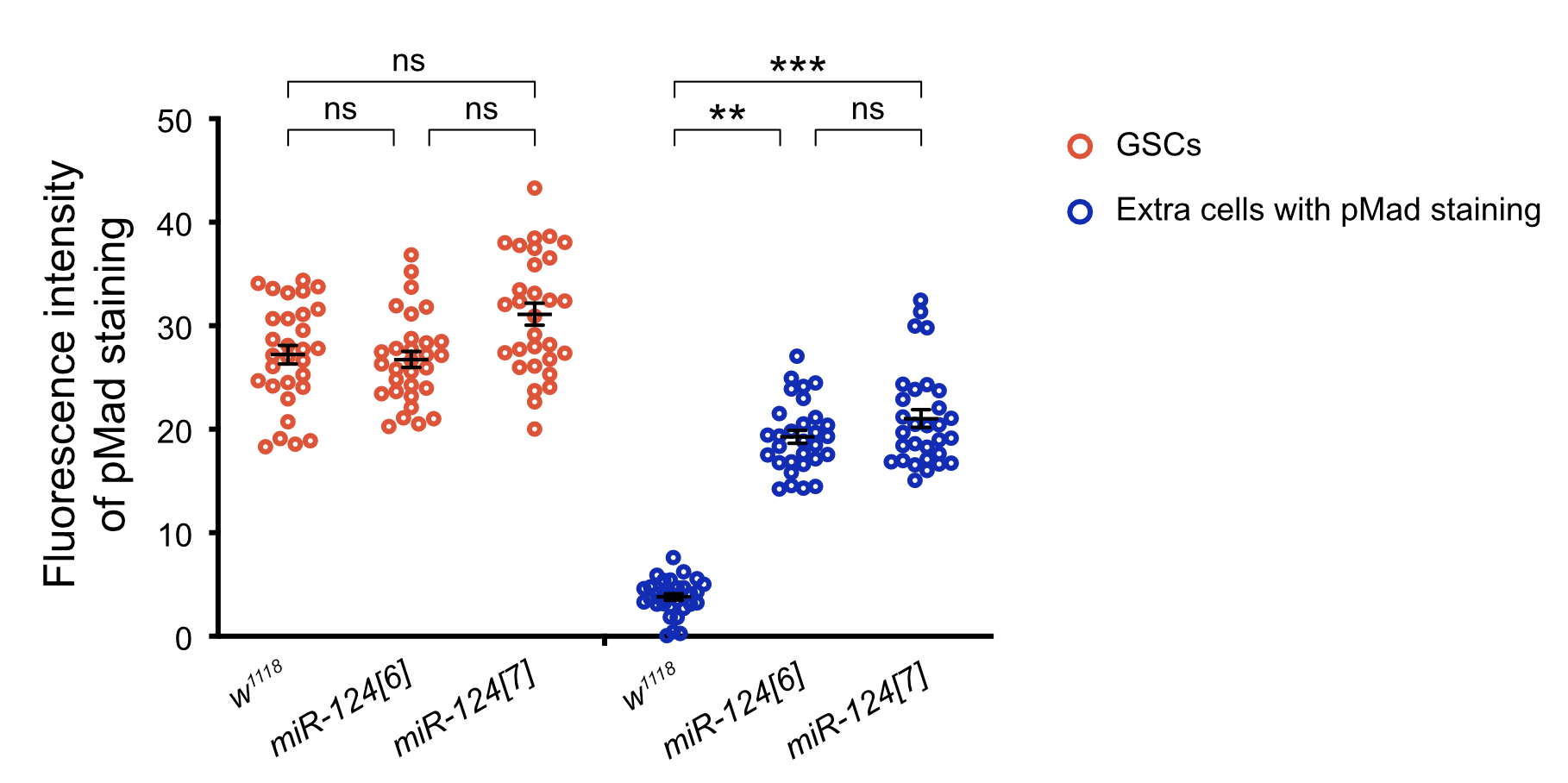

Supplement: S6 Fig — Quantification results on pMad expression in GSCs (red circles) or extra cells (blue circles) per germarium of wild-type, miR-124[6], and miR-124[7] mutants. In wild-type, there are no extra cells with pMad staining, so we choose the cells close to the GSCs for analysis. Each plot indicates the mean intensity from GSCs or extra cells with pMad staining for each germarium (n = 30 Drosophila germaria were examined for each group). Data are presented as the mean ± SEM. Significance was determined by Kruskal–Wallis one-way ANOVA with Dunn’s test (** P < 0.01; *** P < 0.001; ns: not significant). The raw data are available in S1 Data. (TIFF) [file pbio.3002515.s006.tiff]

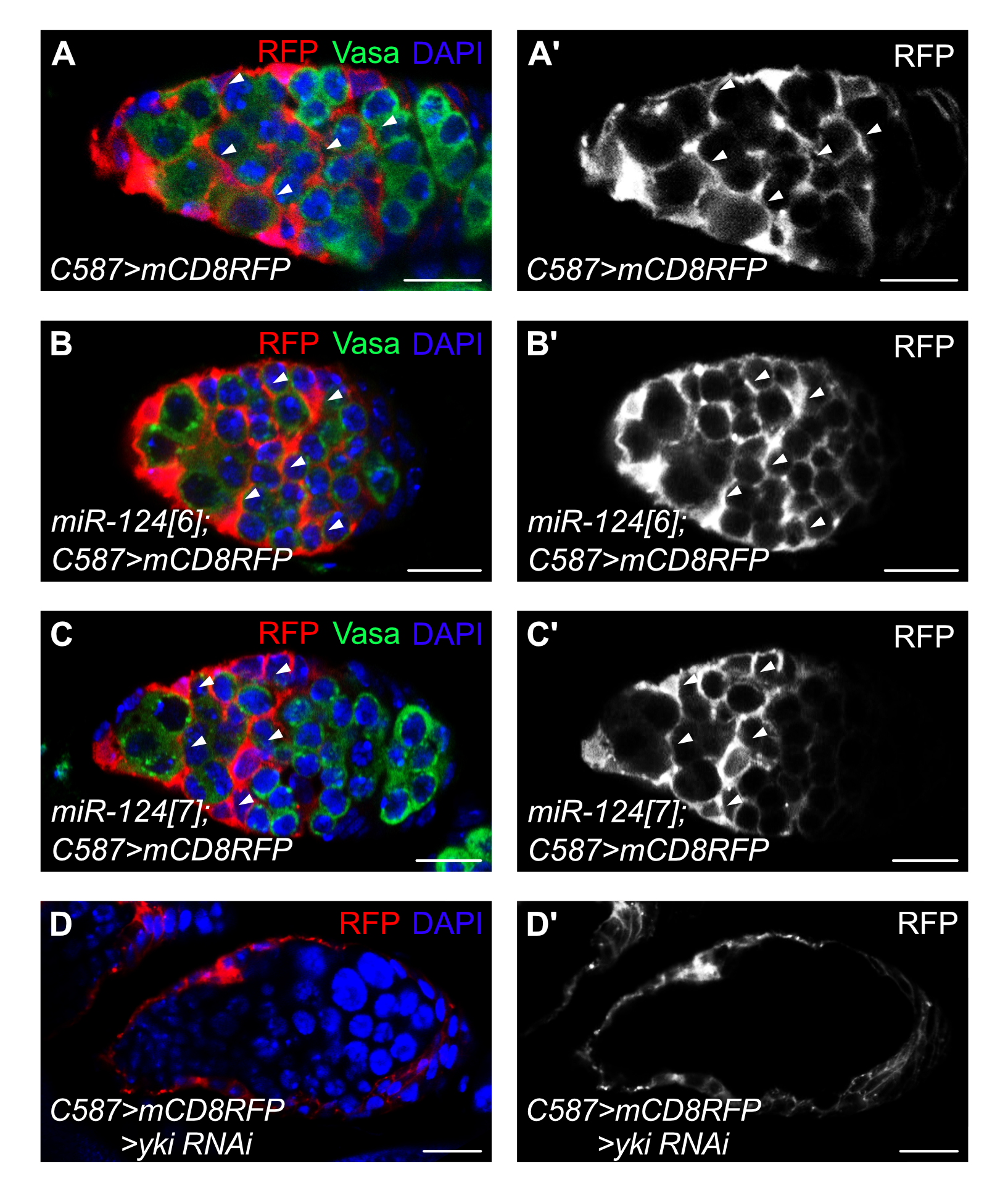

Supplement: S7 Fig — (A–C’) UAS-mCD8RFP and C587-GAL4 were used to indicate EC cellular processes (red) in the germaria of wild-type, miR-124[6], and miR-124[7] mutants. Similar to the control germarium (A–A’), differentiated germ cell cysts (labeled by Vasa, green) were normally wrapped by EC cellular processes in both miR-124[6] (B–B’) and miR-124[7] (C–C’) mutant germaria. (A), (B), and (C) show the merging of the 3 channels of RFP, Vasa, and DAPI (blue); (A’), (B’), and (C’) show RFP stained images in black and white. (D–D’) UAS-mCD8RFP driven by C587-GAL4-labeled EC membranes in yki RNAi germaria. EC cellular processes did not penetrate the interior in yki mutant germaria. (D) Shows the merging of the 2 channels of RFP and DAPI (blue); (D’) shows RFP stained images in black and white. Scale bar: 10 μm. (TIFF) [file pbio.3002515.s007.tiff]

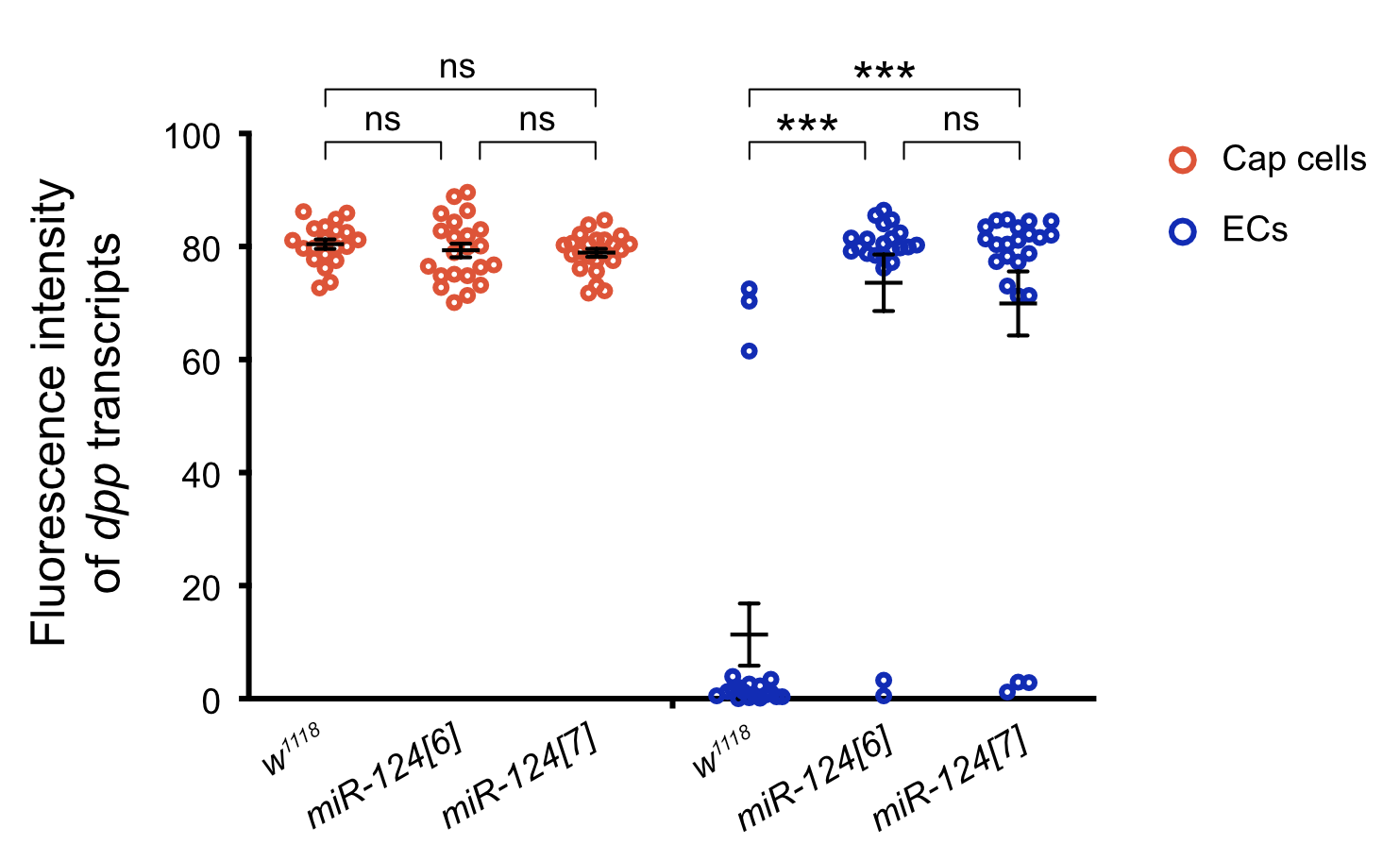

Supplement: S8 Fig — Quantification results on dpp signals in Cap cells (red circles) and ECs (blue circles) per germarium of wild-type, miR-124[6], and miR-124[7] mutants. Each plot indicates the mean intensity from Cap cells or ECs for each germarium. Data are presented as the mean ± SEM. Significance was determined by Kruskal–Wallis one-way ANOVA with Dunn’s test (*** P < 0.001; ns: not significant). The raw data are available in S1 Data. (TIFF) [file pbio.3002515.s008.tiff]

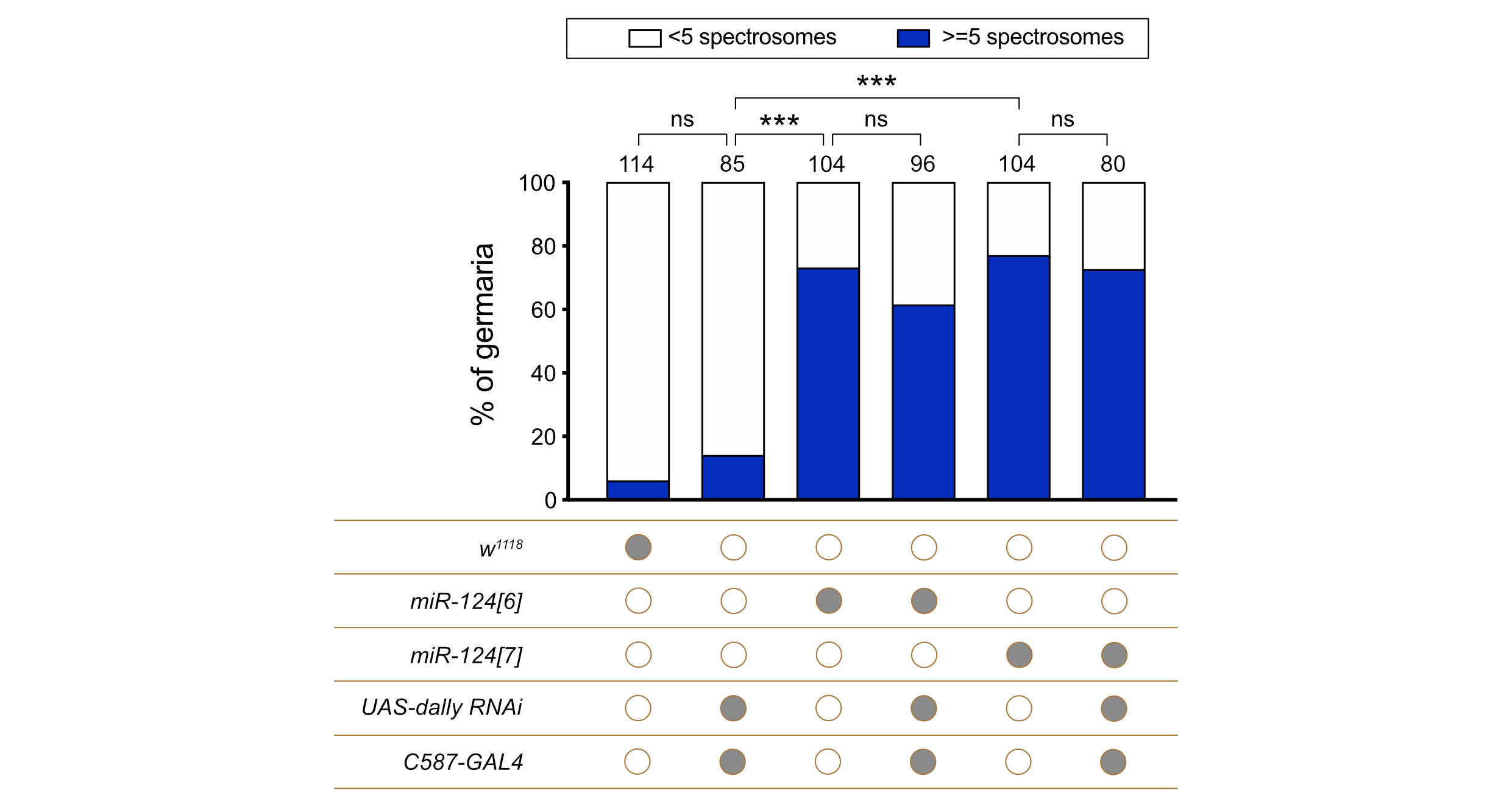

Supplement: S9 Fig — Quantification results on the percentage of the germaria carrying 5 or more spectrosome-containing cells with different genotypes. The number of analyzed germaria is shown above each bar. Significance was determined by Fisher’s exact two-sided test (*** P < 0.001; ns: not significant). Filled gray circles represent the presence, and empty brown circles represent the absence, of a given transgene. The raw data are available in S1 Data. (TIFF) [file pbio.3002515.s009.tiff]

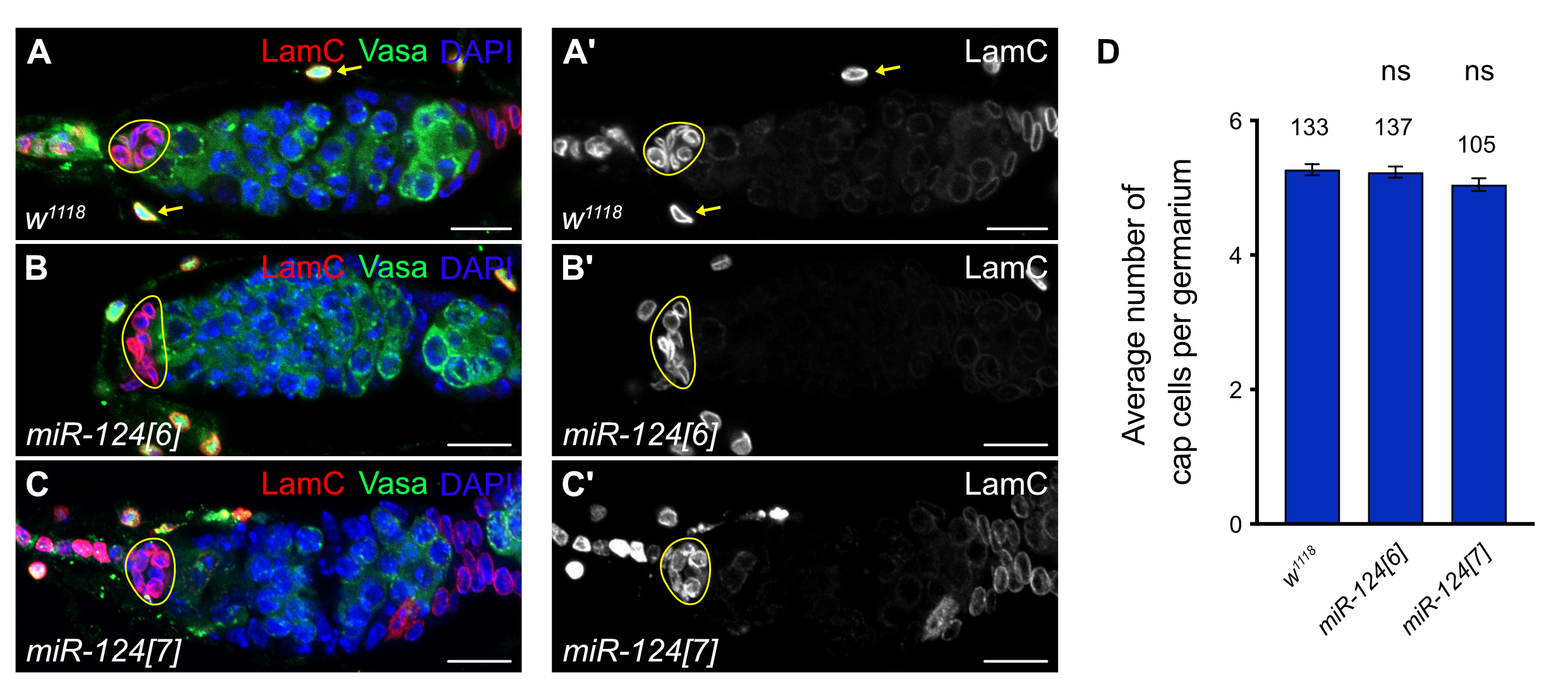

Supplement: S10 Fig — (A–C’) Germaria from wild-type females (A–A’, w1118), miR-124[6] (B–B’), and miR-124[7] (C–C’) mutants were immunostained for LamC (red) to identify Cap cells. Both in the control and miR-124 mutant germaria, LamC is restrictedly expressed in the TF cells, Cap cells (yellow flat circle), and occasionally some muscle sheath cells (yellow arrow) but is not present in ECs. (A), (B), and (C) show the merging of the 3 channels of LamC, Vasa (green), and DAPI (blue); (A’), (B’), and (C’) show LamC stained images in black and white. Scale bar: 10 μm. (D) Quantification results of the average numbers of Cap cells per germarium. Control germaria contain an average number of 5.27 Cap cells in 133 samples; the miR-124[6] and miR-124[7] mutant germaria contain an average number of 5.23 (n = 137) and 5.05 (n = 105) Cap cells, respectively. Data are presented as the mean ± SEM. Significance was analyzed by Kruskal–Wallis one-way ANOVA with Dunn’s test (ns: not significant). The raw data underlying panel D are available in S1 Data. (TIFF) [file pbio.3002515.s010.tiff]

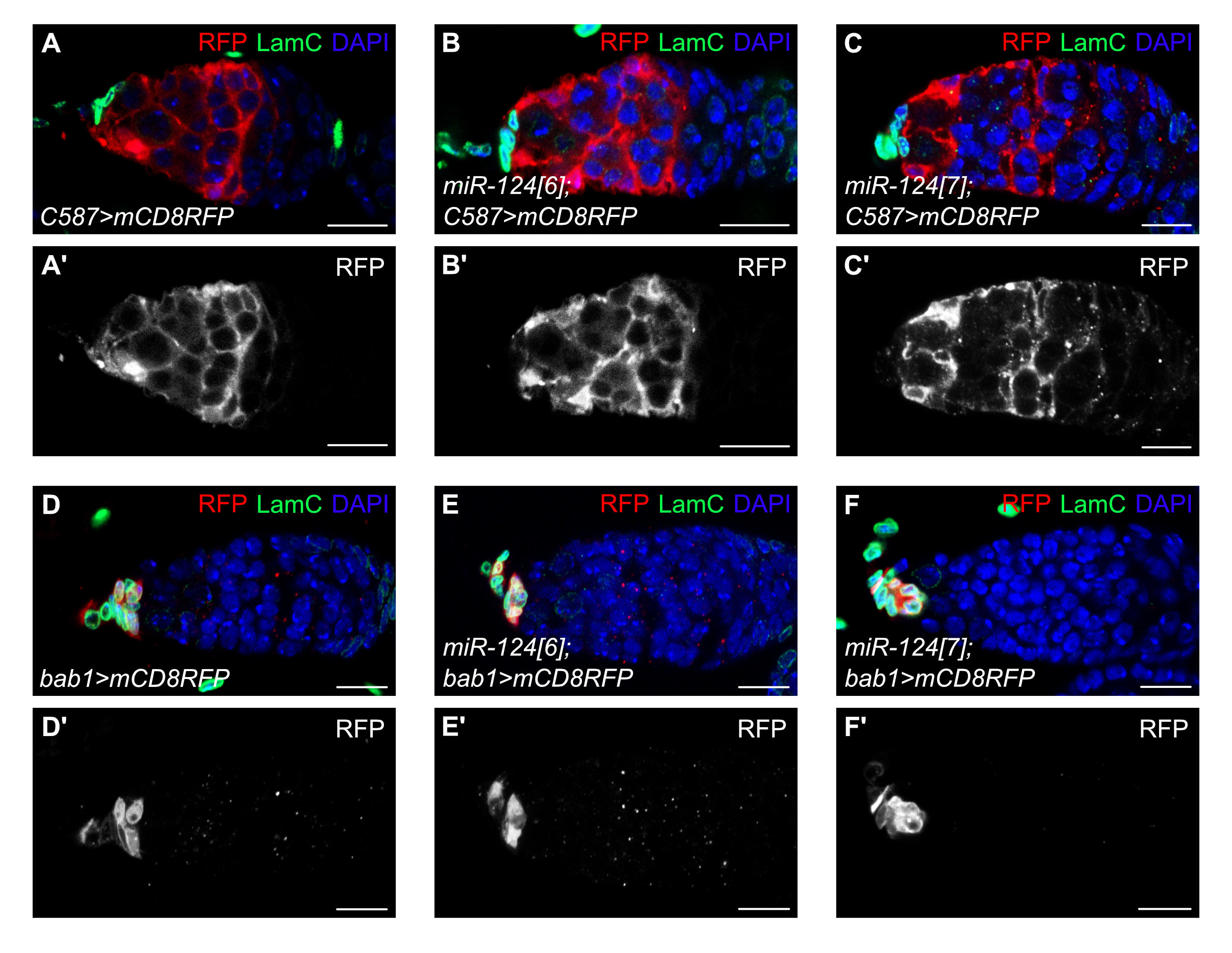

Supplement: S11 Fig — (A–C’) UAS-mCD8RFP and C587-GAL4 were used to indicate the C587-GAL4 expression pattern (red) in the germaria of wild-type (A), miR-124[6] (B), or miR-124[7] (C) mutants. In both the control and miR-124 mutant germaria, no ectopic expression of C587-GAL4 was observed in Cap cells or TF cells (stained with LamC). (D–F’) UAS-mCD8RFP and bab1-GAL4 were used to indicate the bab1-GAL4 expression pattern (red) in the anterior germaria of wild-type (D), miR-124[6] (E), or miR-124[7] (F) mutants, which were also immunostained for LamC (green) to identify Cap cells. Similar to that of the control germarium (D–D’), the expression pattern of bab1-GAL4 was unchanged in both the miR-124[6] (E–E’) and miR-124[7] (F–F’) mutant germaria. (A–F) Show the merging of the 3 channels of RFP, LamC (green), and DAPI (blue); (A’–F’) show RFP stained images in black and white. Scale bar: 10 μm. (TIFF) [file pbio.3002515.s011.tiff]

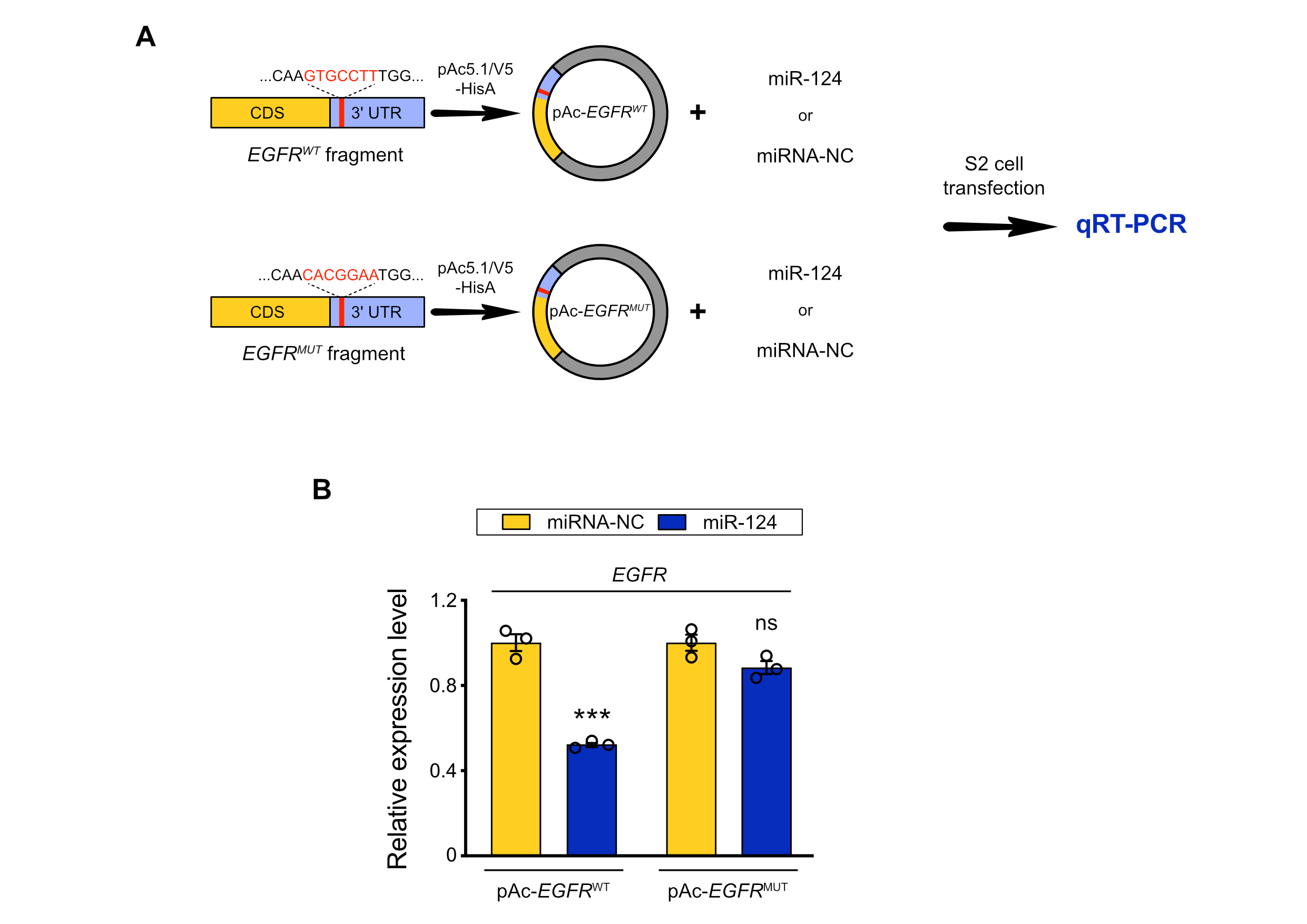

Supplement: S12 Fig — (A) The schematic diagram of recombinant plasmids generation and cell transfections. To generate the recombinant plasmid (pAc-EGFRWT), the sequence of EGFR that contained the coding region and the 3′ UTR fragment was amplified from cDNA of D. melanogaster, and then was cloned into the pAc5.1/V5-HisA insect expression vector (Invitrogen, Cat#V4110-20) at the KpnI site. The mutant construct (pAc-EGFRMUT) with mutation at the binding site of miR-124 was synthesized using Mut Express II Fast Mutagenesis Kit V2 (Vazyme, Cat#C214-02). Drosophila Schneider 2 (S2) cells were co-transfected with the plasmid expression vectors (pAc-EGFRWT or pAc-EGFRMUT) and miR-124 or miRNA-NC at a 1:1 ratio using the Lipofectamine 3000 reagent (Invitrogen, Cat#L3000015) according to the manufacturer’s instructions. All primers used for vector construction are listed in S5 Table. (B) The mRNA expression levels of EGFR were determined in S2 cells co-transfected with the plasmid expression vectors (pAc-EGFRWT or pAc-EGFRMUT) and miR-124 or miRNA-NC using qPCR. All primers used for vector construction are listed in S5 Table. Three biological replicates were performed. Data are presented as the mean ± SEM; significance was analyzed by two-way ANOVA with Sidak’s multiple comparisons test (*** P < 0.001; ns: not significant). The raw data underlying panel B are available in S1 Data. (TIFF) [file pbio.3002515.s012.tiff]

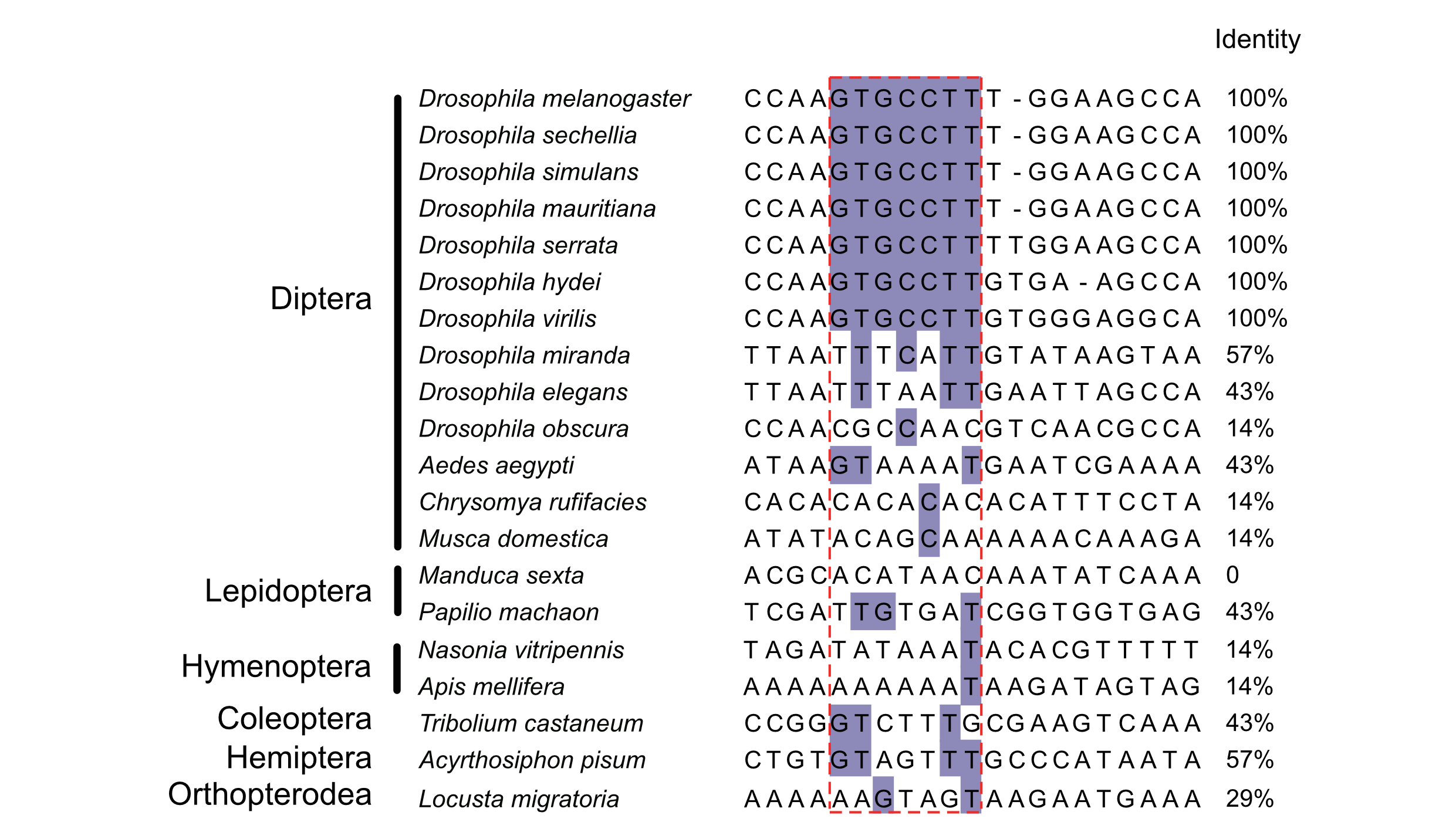

Supplement: S13 Fig — The EGFR 3′ UTR in 20 insect species represent 6 orders, including Diptera (13), Lepidoptera (2), Hymenoptera (2), Coleoptera (1), Hemiptera (1), and Orthopterodea (1) were collected. The alignment results showed that the target sites of miR-124 in EGFR 3′ UTR are conserved in many Drosophila species, but have low or no similarity to other insects. (TIFF) [file pbio.3002515.s013.tiff]

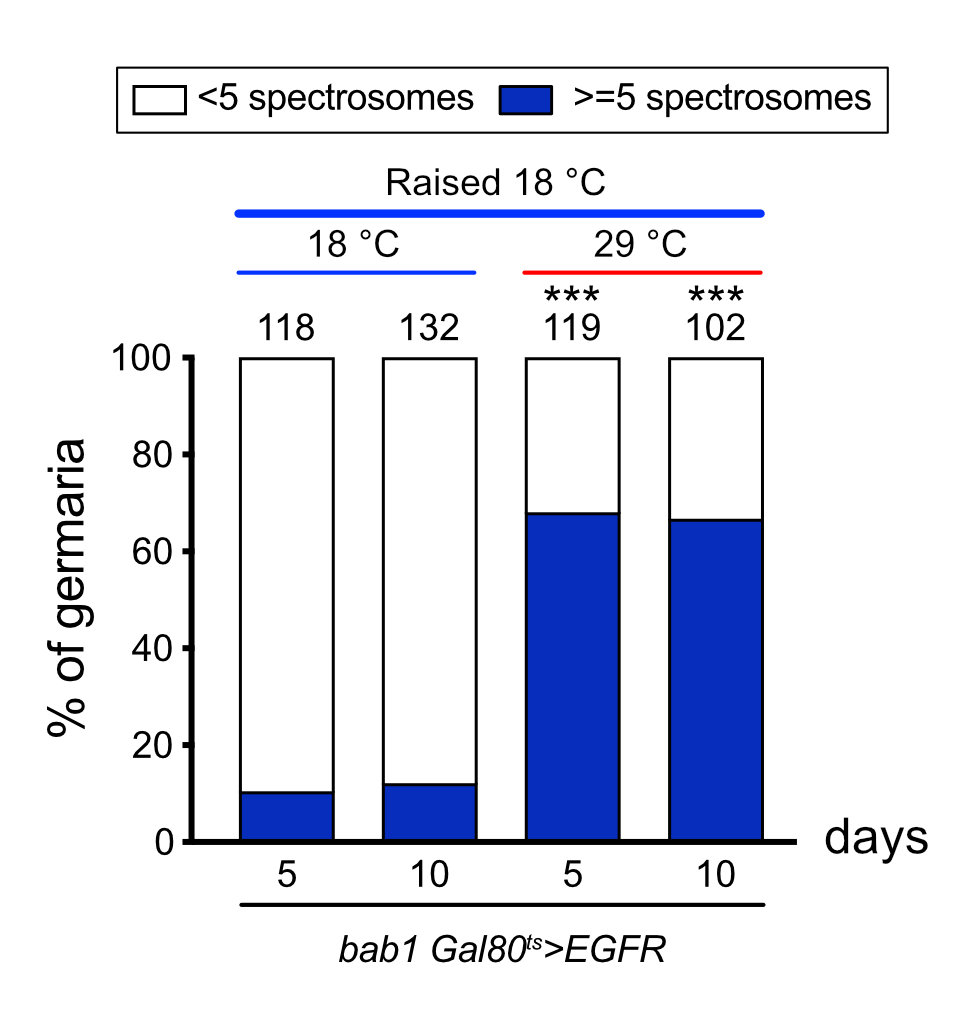

Supplement: S14 Fig — Animals carrying UAS-EGFR and GAL80ts; bab1-GAL4 were raised at 18°C up to eclosion and then maintained at 18°C or 29°C for the number of days indicated before ovary dissection. The percentage of germaria carrying 5 or more spectrosome-containing cells is shown, and the number of analyzed germaria is above each bar. Significance of 18°C vs. 29°C for the same time period was determined by Fisher’s exact two-sided test (*** P < 0.001). The raw data are available in S1 Data. (TIFF) [file pbio.3002515.s014.tiff]

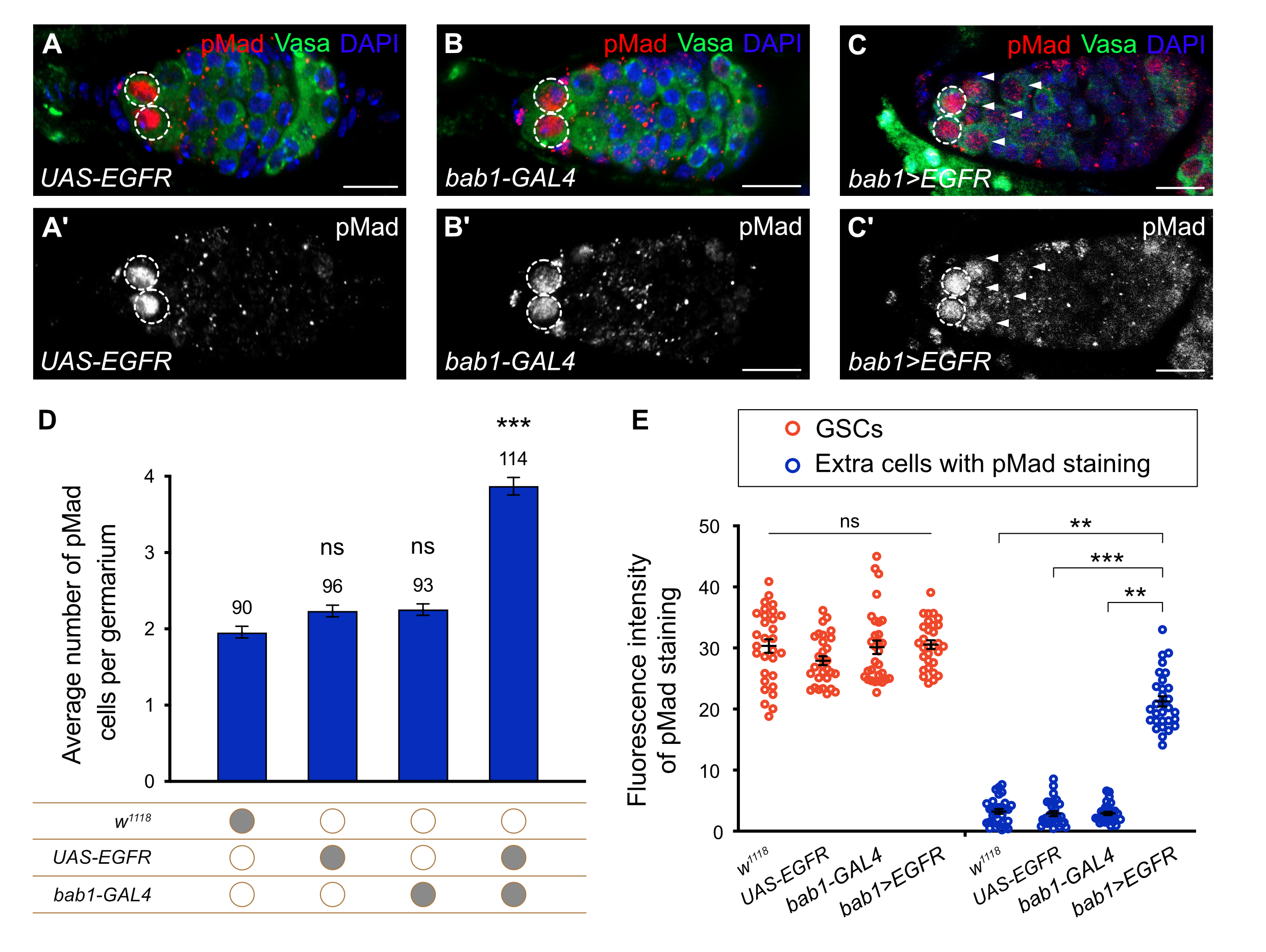

Supplement: S15 Fig — (A–C’) Compared with the control germaria (A–B’, UAS-EGFR or bab1-GAL4) that contained pMad-labeled GSCs only (highlighted by dashed circles), more pMad (red)-positive cells (indicated by arrowheads) were present in bab1-GAL4-derived EGFR-overexpressing germaria (C–C’). (A), (B), and (C) show the merging of the 3 channels of pMad, Vasa, and DAPI (blue); (A’), (B’), and (C’) show pMad stained images in black and white. Scale bar: 10 μm. (D) Quantification results of the average numbers of cells with pMad staining per germarium in different phenotypes. Germaria of w1118 (n = 90), UAS-EGFR (n = 96), or bab1-GAL4 (n = 93) contained average numbers of 1.96, 2.24, and 2.26 pMad cells, respectively. In contrast, the overexpression of EGFR in Cap cells caused ectopic pMad staining in the GSC progeny differentiation zone, with an average number of 3.88 in 114 samples. Data are presented as the mean ± SEM. Significance was analyzed by Kruskal–Wallis one-way ANOVA with Dunn’s test (*** P < 0.001; ns: not significant). Filled gray circles represent the presence, and empty brown circles represent the absence, of a given transgene. (E) Quantification results on pMad expression in GSCs (red circles) or extra cells (blue circles) per germarium of wild-type, UAS-EGFR, bab1-GAL4, and bab1>EGFR mutants. Each plot indicates the mean intensity from GSCs or extra cells with pMad staining for each germarium (n = 30 Drosophila germaria were examined for each group). Data are presented as the mean ± SEM. Significance was determined by Kruskal–Wallis one-way ANOVA with Dunn’s test (** P < 0.01; *** P < 0.001; ns: not significant). The raw data underlying panels D and E are available in S1 Data. (TIFF) [file pbio.3002515.s015.tiff]

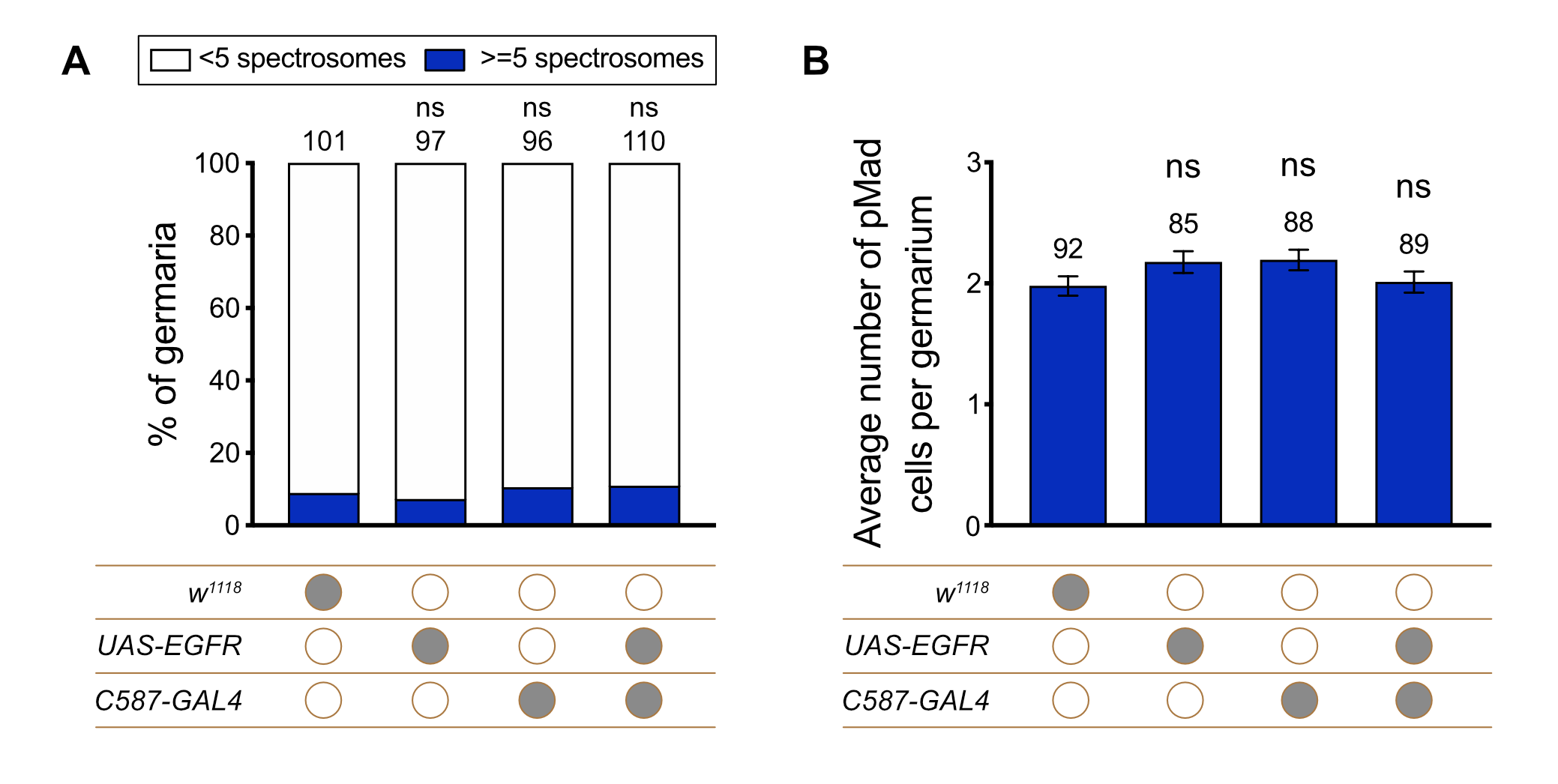

Supplement: S16 Fig — (A) Percentage of the germaria carrying 5 or more spectrosome-containing cells with different genotypes. The number of analyzed germaria is shown above each bar. Significance was determined by Fisher’s exact two-sided test (ns: not significant). (B) Quantification results of the average numbers of cells with pMad staining per germarium in different phenotypes. Germaria of w1118 (n = 92), UAS-EGFR (n = 85), or C587-GAL4 (n = 88) contained average numbers of 1.98, 2.18, and 2.19 pMad cells, respectively. Similarly, the overexpression of EGFR in ECs caused no effect on ectopic pMad staining in the GSC progeny differentiation zone, with an average number of 2.01 in 89 samples. Data are presented as the mean ± SEM. Significance was analyzed by Kruskal–Wallis one-way ANOVA with Dunn’s test (ns: not significant). Filled gray circles represent the presence, and empty brown circles represent the absence, of a given transgene. The raw data are available in S1 Data. (TIFF) [file pbio.3002515.s016.tiff]

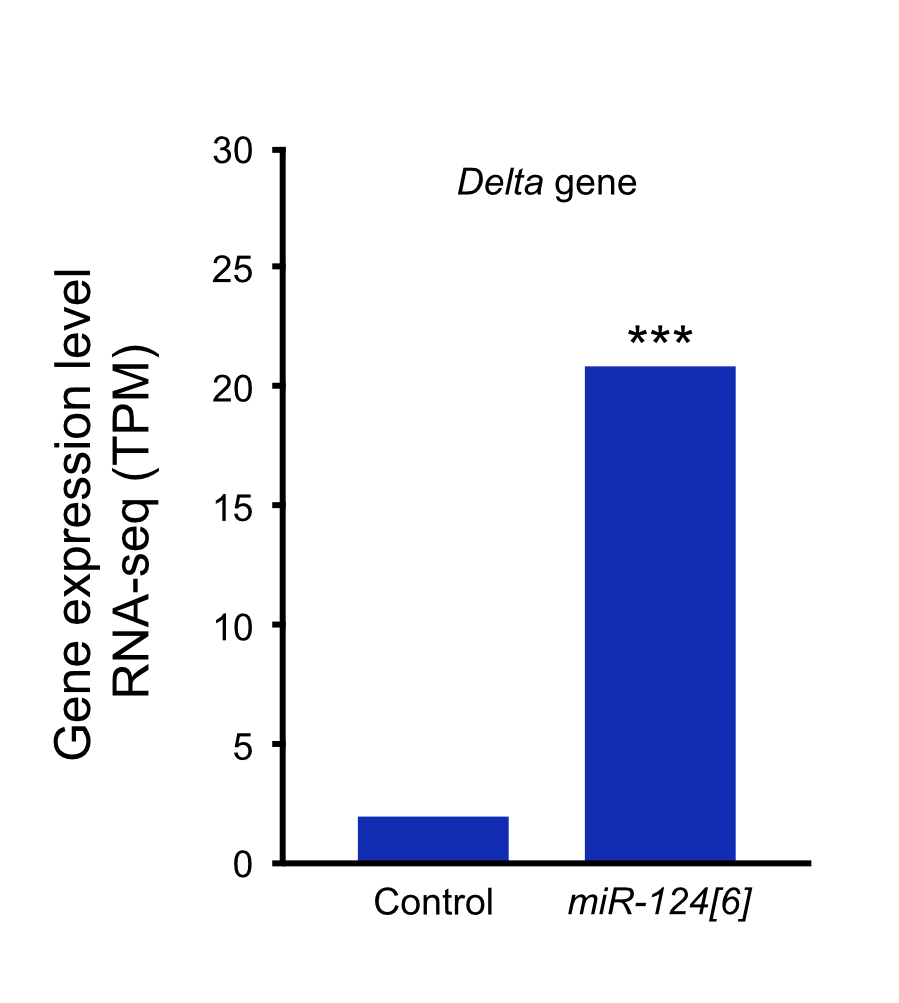

Supplement: S17 Fig — The raw data are available in S1 Data. (TIFF) [file pbio.3002515.s017.tiff]

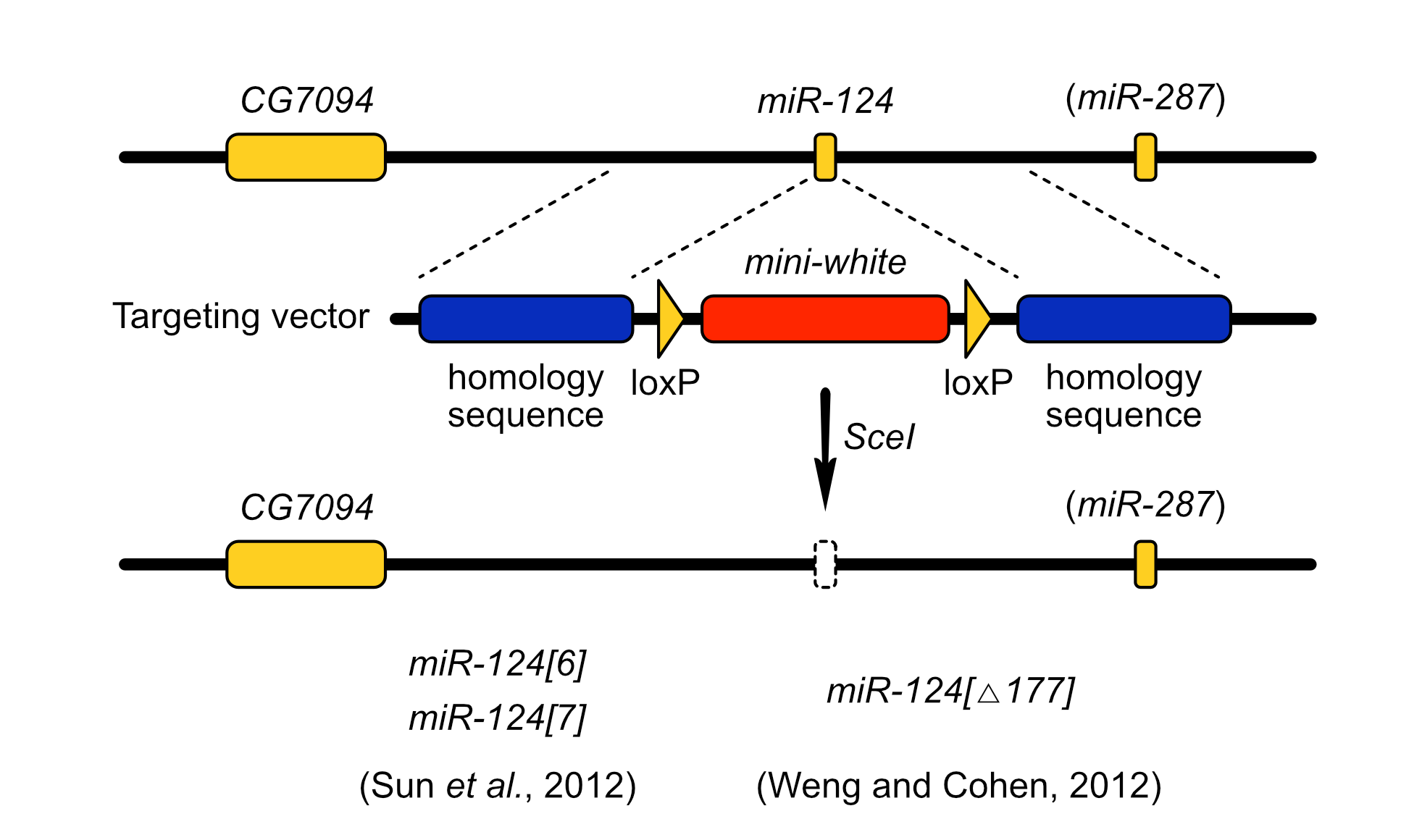

Supplement: S18 Fig — The schematic drawing of miR-124 locus was modified from Sun and colleagues [48] and Weng and Cohen [49]. (TIFF) [file pbio.3002515.s018.tiff]
