# Supplementary material for: Communication between the stem cell niche and an adjacent differentiation niche through miRNA and EGFR signaling orchestrates exit from the stem cell state in the Drosophila ovary
Source: PLoS Biol. 2024 Mar 21;22(3):e3002515. doi: 10.1371/journal.pbio.3002515 (PMC10986965; doi:10.1371/journal.pbio.3002515)
Supplement: Attachment — Submitted filename: Response to Reviewers.docx [file pbio.3002515.s025.docx]

Reviewer #1: In this work, the authors study the transition from an undifferentiated stem cell to a differentiating progeny using the Drosophila ovarian niche as their model system. They report that miR-124 acts to repress EGFR transcription in some somatic cells of the niche (terminal filament cells and cap cells). Increased EGFR signaling in miR-124 mutant niche cells induced Delta (one of the Notch ligands) transcription. Reception of the Dl signal by Notch in the escort cells activates/enhances dpp transcription, thus preventing germline differentiation.

I believe the biological question under research to be of wide interest. The MS is well written and the results are carefully documented. I do however have some concerns regarding the conclusions reached by the AUs.

**AUTHORS’ RESPONSE: We appreciate that the reviewer thought the study was interesting, well-written and that conclusions were carefully documented. We also thank the reviewer for the suggestions on how to improve it.**

1-The use of the bab1-Gal4 line to address TF + cap cell versus escort cell realm of action. The data presented by the authors seem to indicate that the bab1-Gal4 line used in this study is expressed in TF and cap cells only. The authors cite a number of previous publications that reinforce their findings. There are in the literature however contradictory results regarding this line (which the authors do not even acknowledge), with some authors unequivocally showing that some bab1-Gal4 lines are expressed clearly in escort cells. In fact, some of the publications used by the authors to confirm the bab1-Gal4 expression in TF and cap cells only do show/mention that this line is also expressed in escort cells (see, for instance, Bolivar et al 2006; Hamada-Kawaguchi 2014). Since this is a key point in this work (is miR-124 acting in the escort cells in addition to TF and cap cells?) I believe the authors should check with a TF and cap cell specific line the validity of their results. This is particularly important considering (1) that miR-124 is expressed in the most anterior escort cells too, (2) the data in Fig. 2D and (3) my concern number 2.

**AUTHORS’ RESPONSE: We thank the reviewer for pointing out this important issue about the specificity of the *bab1-GAL4* line. We carefully investigated the literature for *bab1-GAL4* lines and their reported expression patterns. We found that the first *bab1-GAL4* lines were generated by the Laski lab (Cabrera et al., 2002), which were bab*^Agal4-5^* and bab*^Pgal4-2^*. The two lines have the same expression pattern, reported as expression only in terminal filament (TF) cells and Cap cells. The two lines were available from the Bloomington *Drosophila* Stock Center (BL#6802 for bab*^Agal4-5^* and BL#6803 for bab*^Pgal4-2^*), and were widely used by other labs (See the table below). The reported expression specificity of bab*^Agal4-5^* and bab*^Pgal4-2^* has been confirmed by several independent labs (See the table below), and, most importantly for this study, by our own careful analysis (S4A and S4A’ Figs).**

| Authors | Title | Year | Journal | Expression pattern described by authors | Note |
| --- | --- | --- | --- | --- | --- |
| Cabrera et al. | Expression pattern of Gal4 enhancer trap insertions into the *bric à brac* locus generated by P element replacement. | 2002 | *Genesis* | Stainings of adult ovaries show that both *bab^Agal4-5^* and *bab^Pgal4-2^* express Gal4 at high levels in the terminal ﬁlament cells, and this was the predominant staining pattern in the adult ovary. | This is the first report for bab1-Gal4 lines, which were generated by Cabrera and Laski et al. in 2002 (this study). The two lines have the same expression pattern and was available in Bloomington *Drosophila* Stock Center (BL#6802 for *bab^Agal4-5^* and BL#6803 for *bab^Pgal4-2^*).    The staining (LacZ) results of the two bab1-Gal4 lines |
| Park et al. | The miRNA pathway intrinsically controls self-renewal of *Drosophila* germline stem cells | 2007 | *Current Biology* | P{bab-Gal4} line was used to drive cell lineage-speciﬁc expression of terminal ﬁlament and cap cells. | The authors used *bab^Agal4-5^* line (BL#6802) in this study, and they confirmed the specificity of bab1-Gal4 expression through driving UAS-GFP.   |
| Guo & Wang | The glypican Dally is required in the niche for the maintenance of germline stem cells and short-range BMP signaling in the *Drosophila* ovary | 2009 | *Development* | Bab1Gal4 is strong in both TF and cap cells… | The authors used *bab^Pgal4-2^* line (BL#6803) in this study, and they confirmed the specificity of bab1-Gal4 expression through driving UAS-GFP. However, the authors have a wrong citation (Bolívar et al., 2006). The correct citation should be Cabrera et al. 2002.  |
| Sahai-Hernandz & Nystul | A dynamic population of stromal cells contributes to the follicle stem cell niche in the *Drosophila* ovary | 2013 | *Development* | Bab1-Gal4-5 is commonly used to express transgenes specifically in terminal filament cells and cap cells. | The authors used *bab^Agal4-5^* line (BL#6802) in this study, and they confirmed the specificity of bab1-Gal4 expression through driving UAS-GFP.   |
| Liu et al. | Coordinated niche-associated signals promote germline homeostasis in the *Drosophila* ovary | 2015 | *Journal of Cell Biology* | …and Bab1 (which drives reporter expression in TF and cap cells) ... | The authors used *bab^Agal4-5^* line (BL#6802) in this study. |
| Luo et al. | Wnt ligands regulate Tkv expression to constrain Dpp activity in the *Drosophila* ovarian stem cell niche | 2015 | *Journal of Cell Biology* | ...Bab1-gal4, which is expressed in terminal filament cells and cap cells. | The authors used *bab^Agal4-5^* line (BL#6802) in this study. |
| Luo et al. | Engrailed acts with Nejire to control *decapentaplegic* expression in the *Drosophila* ovarian stem cell niche | 2017 | *Development* | …we used the bab1-gal4 driver, which is expressed in TF and cap cells, … | The authors used *bab^Agal4-5^* line (BL#6802) in this study. |
| Kim-Yip & Nystul | Wingless promotes EGFR signaling in follicle stem cells to maintain self-renewal | 2018 | *Development* | ...using the cap and terminal filament cell driver, bab1-Gal4, ... | The authors used both *bab^Agal4-5^* line (BL#6802) and *bab^Pgal4-2^* line (BL#6803) in this study. |
| Kober et al. | Loss of putzig in the germline impedes germ cell development by inducing cell death and new niche like microenvironments | 2019 | *Scientific Reports* | … with the cap cell specific driver *bric à brac* (bab1)-Gal4, ... | The authors used *bab^Agal4-5^* line (BL#6802) in this study. |
| Zhu et al. | Piwi in the stem cell niche regulates nurse cell number and oocyte specification | 2020 | *Micropublication Biology* | Bab1-GAL4 driving UASp-GFP demonstrates that the driver is expressed in terminal filament and cap cells. | The authors used *bab^Agal4-5^* line (BL#6802) in this study, and they confirmed the specificity of bab1-Gal4 expression through driving UAS-GFP.   |
| Zhao et al. | Notch signaling governs the expression of glypican Dally to define the stem cell niche | 2020 | *Biology open* | …, bab1-GAL4, active in cap cells and terminal filaments, ... | The authors used *bab^Pgal4-2^* line (BL#6803) in this study, but have a wrong citation (Bolívar et al., 2006). The correct citation should be Cabrera et al. 2002. |
| Almeida-Oliveira et al. | dHNF4 regulates lipid homeostasis and oogenesis in *Drosophila melanogaster* | 2021 | *Insect Biochemistry and Molecular Biology* | The localization of bab1GAL4 expression is Terminal filaments and cap cells. | The authors used *bab^Agal4-5^* line (BL#6802) in this study. |
| Taniguchi & Igaki | Sas-Ptp10D shapes germ-line stem cell niche by facilitating JNK-mediated apoptosis | 2023 | *PLoS Genetics* | ...bab1-Gal4, which drives gene expression at the larval stage in apical cells, terminal filament cells, and swarm cells, as well as at the adult stage in terminal filament cells and cap cells… | The authors used *bab^Agal4-5^* line (BL#6802) in this study. |

**There is another *bab1-GAL4* line which was independently generated by the Gonzalez-Reyes lab (Bolívar et al., 2006). The expression pattern of this line is reported as slightly different, with high levels in adult TFs and Cap cells but also with weaker expression in some ECs. Though this line was not available in any *Drosophila* Stock Centers, it was used for in other labs (See the table below). The reported expression pattern of this *bab1-GAL4* line (Bolívar et al., 2006) was confirmed by several labs. This line was nevertheless treated as a TFs and Cap cell-speciﬁc *GAL4* driver in some studies (See the table below).**

| Authors | Title | Year | Journal | Expression pattern described by authors | Note |
| --- | --- | --- | --- | --- | --- |
| Bolívar et al. | Genetic dissection of a stem cell niche: the case of the *Drosophila* ovary | 2006 | *Developmental Dynamics* | In adult bab1-Gal4 ovaries, we observed strong UAS-nod:GFP expression in TFCs and CpCs; We also detected GFP expression in anterior IGS cells, which on the basis of their localization and morphology, represent ESCs and Ecs. | This bab1-Gal4 line was independently generated by Bolívar and Gonza´ lez-Reyes et al. in 2006 (this study). This line was NOT available in any *Drosophila* Stock Centers, and was duplicated in some fly labs for different studies.    The anti-GFP staining results of bab1-Gal4 driving UAS-GFP expression. |
| López-Onieva et al. | Jak/Stat signalling in niche support cells regulates dpp transcription to control germline stem cell maintenance in the *Drosophila* ovary | 2008 | *Development* | ...using the bab1-Gal4 line, which induces strong expression of reporter genes in TFCs and CpCs and weaker levels in ESCs and ECs. | The authors used the bab1-Gal4 line from Gonza´ lez-Reyes lab (Bolívar et al., 2006), and they confirmed the bab1-Gal4 expression through driving DsRed.   |
| Rojas-Ríos et al. | Cytoneme-mediated delivery of Hedgehog regulates the expression of bone morphogenetic proteins to maintain germline stem cells in *Drosophila* | 2012 | *PLoS Biology* | Thus, we utilised the bab1-Gal4 driver to express modified versions of two known regulators of actin polymerization in TFCs and CpCs of adult ovaries. | The authors used the bab1-Gal4 line from Gonza´ lez-Reyes lab (Bolívar et al., 2006). However, They used this line to express genes in terminal filaments (TFCs) and cap cells (CpCs). |
| Hamada-Kawaguchi et al. | Btk29A promotes wnt4 signaling in the niche to terminate germ cell proliferation in *Drosophila* | 2014 | *Science* | ...via bab1-Gal4 , which showed high levels of expression in terminal filament cells and cap cells and lower levels of expression in escort cells. | The authors did NOT confirm the expression pattern of the bab1-Gal4 line they used in the study. They also did NOT provide the information where they got this bab1-Gal4 line. They might get the line from Gonza´ lez-Reyes lab (Bolívar et al., 2006). |
| Bonfini et al. | Reversible regulation of stem cell niche size associated with dietary control of Notch signalling | 2015 | *BMC Developmental Biology* | The bric-a-brac1-Gal4 (Bab1-Gal4) drives expression in cap cells, in the terminal filament and more weakly in the anterior escort cells. | The authors used the bab1-Gal4 line from Gonza´ lez-Reyes lab (Bolívar et al., 2006). |
| Ayyub et al. | Reduction of Cullin-2 in somatic cells disrupts differentiation of germline stem cells in the *Drosophila* ovary | 2015 | *Developmental Biology* | … using bric-a-brac1 (bab1)-Gal4, a CpCs and TFCs-speciﬁc Gal4 driver with a weak expression in ECs. | The authors used the bab1-Gal4 line from Gonza´ lez-Reyes lab (Bolívar et al., 2006). The authors confirmed the bab1-Gal4 expression through driving UAS-GFP, and they used this line as a CpCs and TFCs-speciﬁc Gal4 driver.   |
| Lu et al. | COP9-Hedgehog axis regulates the function of the germline stem cell progeny differentiation niche in the *Drosophila* ovary | 2015 | *Development* | …bab1-gal4 is widely used to drive and knock out gene expression in CPCs. | The authors used the bab1-Gal4 line from Gonza´ lez-Reyes lab (Bolívar et al., 2006), but they believe that bab1-gal4 is widely used to drive and knock out gene expression in CPCs. |
| Lobo-Pecellín et al. | Mastermind regulates niche ageing independently of the Notch pathway in the *Drosophila* ovary | 2019 | *Open biology* | bab1-Gal4 is expressed at high levels in adult TFCs and CpCs; it is also expressed, albeit weakly, in Ecs. | The authors used the bab1-Gal4 line from Gonza´ lez-Reyes lab (Bolívar et al., 2006). |

**In summary, there are two types of *bab1-GAL4* lines from different origins. It seems that they have slightly different expression pattern. In our study, we used bab*^Agal4-5^* line (BL#6802). We looked very carefully at the expression pattern of the line we used, both in the wild-type condition (S4 Fig) and in *miR-124* mutant samples (S11 Fig) and found very robust signal in TF and Cap cells with no hint of signal in ECs. We believe that these direct results, together with an explanation of why other publications find expression in ECs from a different *bab1-GAL4* line should settle the understandable initial concern about cellular specificity in our study. To our knowledge, this *bab1-GAL4* line is the best for specific expression experiments in TFs and Cap cells and no other alternatives are available to confirm the results directly. We thank the reviewer for raising this comment, which helped to clarify the origins and the specificity of different *bab1-GAL4* lines. Based on that review of the literature, we have corrected some previously incorrect citations for the *bab1-GAL4* line in our original manuscript.**

**We also thank the reviewer for raising the specific concerns (1) that miR-124 is sometimes expressed in the most anterior escort cells too, and (2) the weak rescue data with *C587-GAL4* in Fig. 2D, both hinting at a possible function in the most anterior ECs. We acknowledge this possibility but we wish to emphasize that miR-124 expression in TF and Cap cells rescues fully, whereas rescue through strong expression in ECs is very low, and that expression of miR-124 in ECs (like EC rescue) is only occasional. We therefore modified the text (lines 198-201) as below:**

“*These results are consistent with miR-124 acting in TF or Cap cells, where miR-124 is strongly expressed, to promote GSC differentiation. It is possible that miR-124 activity in anterior ECs can also sometimes contribute to GSC differentiation, reflecting occasional expression in those cells.*”

**We also understand that the reviewer has thought carefully about the whole picture and hence about other considerations that may challenge our conclusions about where miR-124 (and EGFR) act.**

**The point above (about miR-124 expression in anterior ECs) is one example. As explained above, the major site of action of miR-124 is clearly in TF or Cap cells, not ECs.**

**Another example is the concern that Delta signaling from Cap cells may not extend far enough to induce ectopic *dpp* production in cells not immediately adjacent to Cap cells. We agree that this is an interesting and important consideration. We have therefore taken great care to explain exactly where and how often miR-124 phenotypes affect germline (pMad and differentiation) and EC (dpp RNA) phenotypes. The most prevalent responses are close to Cap cells but there are also more distant responses. We have amended the discussion to repeat these observations and mention several potential explanations, including the possibility that the most anterior EC might occasionally act more like a Cap cell or the possibility that not all responses derive directly from altered Delta-Notch signaling (lines 473-495).**

**Having carefully considered both the bigger picture questions and the data regarding *bab1-GAL4* specificity, we would like to re-iterate that the expression pattern data, and hence the inferences concerning cellular focus of various gene activities, are very strong. Both the *bab1-GAL4* and *C587-GAL4* lines we used are expressed strongly in complementary cell types without any detectable crossover in wild-type and *miR-124* mutant conditions. These are primary data, which we respect as key drivers of our conclusions. We also believe that the series of results we obtain driving expression variously of miR-124, EGFR, EGFR RNAi, Notch RNAi and Dpp are consistent with both the noted expression domains of these *GAL4* lines and the model we put forward.**

2-According to their model, cap cells express miR-124 to repress EGFR transcription (and thus excess activation of the pathway). In fact, EGFR mRNA has a target for miR-124 and knocking down EGFR transcription using bab1-Gal4 in a miR-124 mutant background rescues the extra-spectrosomes phenotype. Increased EGFR activity induces extra Delta in miR-124 mutant cap cells, which in turn signals to adjacent escort cells via the Notch receptor pathway. Notch activation in escort cells (in theory, the ones adjacent to the cap cells) would then upregulate dpp transcription. Allegedly, this increase in dpp would prevent CB differentiation. My problem with this model is that the anterior escort cells are known to be of different quality to more posterior ones, and they express dpp. Thus, are the authors implying in their model that miR-124 mutants express more dpp only in these anterior escort cells? But they show in figure 4 that more posterior miR-124 mutant escort cells also

express dpp. If their model is correct, how does Dl in the cap cells reach to posterior escort cells? I may add that, if miR-124 acts not only in cap cells but also in anterior escort cells (as suggested by their own data, Fig 2D), this could explain why dpp is expressed in more posterior escort cells in the mutant condition. In summary, I am not convinced that the authors have fully proven that miR-124 acts only in TF and cap cells and that the increase in Dl levels in mutant cap cells is responsible for the observed phenotypes.

**AUTHORS’ RESPONSE: This is another important issue. It is true that several reports have shown that the anterior escort cells sometimes express Dpp (Wang et al., 2008; Liu et al., 2010; Wang & Page-McCaw et al., 2018). The most careful quantitation of *dpp* expression was done in Wang & Page-McCaw (2018). In their findings, only 13% of control germaria had a clear *dpp* signal in**

**a single anterior escort cell. Consistent with their observations, we found 15% of control germaria had a clear *dpp* signal in escort cells. We then looked carefully at all of our images of ectopic *dpp* expression; we found 0.15 ECs on average have ectopic *dpp* in control germaria, and all in the most anterior ECs (S4 Table). However, we found 90.91% and 86.96% ECs on average have ectopic *dpp* in miR-124[6] and miR-124[7] germaria. Among them, 1.05 and 1.09 on average in anterior (region 1) ECs and 0.36 and 0.48 on average in region 2a ECs (S4 Table). We have described these results in the main text (lines 257-258, 261-265) and new S4 Table.**

**We believe that our very careful exposition of these data lay out the raw data, for which the reviewer requested clarification. Now, to put these data into the context of models there are two distinguishable considerations. First, the expression of *dpp* in anterior ECs is considerably higher and more frequent than wild-type when miR-124 is absent (or EGFR overexpressed). This result (and the excess of spectrosome-containing cells very close to GSCs) are fully consistent with the proposed model and ectopic Dl signaling from Cap cells to anterior ECs. Second, our data clearly show that there are sometimes (albeit less frequently) ECs that are more posterior which either express *dpp* detectably or are adjacent to germline cells with pMad or spectrosomes. We appreciate that this phenomenon raises additional questions (without negating the basic model, which accounts for the major site of ectopic *dpp* production). There are many possible explanations, including the exchange of EC positions over time, a potentially longer range of Dl signaling through various cell processes, a potential indirect consequence of changes initiated in anterior ECs or, as the reviewer suggests, the occasional instance of an anterior EC behaving like a Cap cell. None of these can be ruled out easily and none has been investigated further (in a study that is already extremely extensive). They are all now mentioned in a revised discussion that highlights the issue the reviewer appropriately points out to be highly worthy of discussion.**

**We thought it would not be appropriate to discuss these possibilities in detail. Thus, we do not specifically note that Dl has been found to show long-range signaling ability: Dl can be delivered from primordial germ cells to TFs via cellular processes (Yatsenko & Shcherbata, 2021) and long distance Dl-Notch signaling pathway was inferred to be necessary in *Drosophila* bristle development in wing discs (Moore et al., 2020).**

References:

Wang L, Li Z, Cai Y. The JAK/STAT pathway positively regulates DPP signaling in the *Drosophila* germline stem cell niche. J Cell Biol. 2008;180:721-728.

Liu M, Lim TM, Cai Y. The *Drosophila* female germline stem cell lineage acts to spatially restrict DPP function within the niche. Sci. Signal. 2010;3:ra57.

Wang X, Page-McCaw A. Wnt6 maintains anterior escort cells as an integral component of the germline stem cell niche. Development. 2018;145:dev158527.

Yatsenko AS, Shcherbata HR. Distant activation of Notch signaling induces stem cell niche assembly. PLoS Genet. 2021;17(3):e1009489.

Moore RE, Clarke J, Alexandre P. Protrusion-Mediated Signaling Regulates Patterning of the Developing Nervous System. Front Cell Dev Biol. 2020;8:579073.

3-Figs 1, 2 and/or 3 could be combined in just one or two figures.

**AUTHORS’ RESPONSE: We have combined the original Figs 2 and 3 into a new Fig 2.**

4-Fig. 5: Did the authors find other target genes (apart from the 130 known targets) that were not predicted to be bound by miR-124 (may be indirect targets)?

**AUTHORS’ RESPONSE: As the reviewer suggested, the indirect targets of miR-124 is an interesting question but something we did not examine, and we believe it is beyond the current study’s scope.**

5-Line 66: consider prospective "daughter CB".

**AUTHORS’ RESPONSE: It has been corrected as suggested.**

6-Lines 127, 128: The spectrosome in GSCs is not always spherical. It follows a well-described cycle along the cell cycle.

**AUTHORS’ RESPONSE: We have deleted “spherical”.**

7-Fig. 1H': Consider a black and white version of this panel to enhance the contrast.

**AUTHORS’ RESPONSE: We have updated this panel with a black and white version to enhance the contrast.**

8-Ref. 57: These authors showed bab1-Gal4 expression in escort cells.

**AUTHORS’ RESPONSE: Thank you. We have corrected our mistake by providing the right reference, because our *bab1-GAL4* was generated by Laski lab (Cabrera et al., 2002).**

9-Line 193: Data in Fig 2D indicates that miR-124 does have a function in escort cells. I do not understand why the authors neglect this finding in their conclusions.

**AUTHORS’ RESPONSE: This has been addressed above. We do not ignore this result but we put it in perspective relative to the far greater rescue afforded by *bab1-GAL4*.**

10-Line 200: Why do the authors use reference 60 to cite pMad expression in GSCs? It was reported previously to this 2020 paper. Consider Kai + Spradling 2004 or Zhao et al Ageing Cell 2008.

**AUTHORS’ RESPONSE: We have changed the reference and used Kai & Spradling 2004 paper in the revision.**

11-Legend to Fig S6: substitute mutant for mutation.

**AUTHORS’ RESPONSE: It has been corrected as suggested.**

12-To look at escort cell projections I am not sure that the c587-Gal4 line is the best option. I would suggest the authors to generate MARCM clones with CD8:GFP to look at the shape of individual or small clusters of escort cells in control and mutant conditions.

**AUTHORS’ RESPONSE: We have tested for defects in EC processes by using *C587-GAL4* with *UAS-CD8:RFP* to drive a *yki* RNAi line. Similar to previously published data, the results showed that EC processes were completely absent from the interior of germaria when Yki was downregulated in ECs. This test, now included in the manuscript, shows that using *C587-GAL4* with *UAS-CD8:RFP* can certainly detect drastic changes in escort cell processes. Certainly, there could be subtle defects, which might be visualized when examining single cells (in MARCM). That is far from the focus of our studies and would not influence our key conclusions. We included the *C587>CD8-RFP* result simply to compare to some other genotypes that disrupt germline differentiation and drastically alter EC processes.**

13-Lines 229 and 230: This is slightly confusing. First, as indicated above, it is not clear to me that the focus of the action of miR-124 is in TF and Cap cells. Second, if the authors are referring to an alternative explanation for their 124 results, I would recommend them to rephrase the sentence.

**AUTHORS’ RESPONSE: This comment has been addressed above; and we have re-phrased in the text (lines 241-244).**

“*Here, the major focus of action of miR-124 is in TF and Cap cells rather than ECs and ectopic BMP signaling is limited to anterior regions. We therefore investigated whether Dpp or Dally production might be altered by miR-124, and in which cells this might occur*”

14-Lines 264 and 265: Again, I would be more cautious about the specificity issue.

**AUTHORS’ RESPONSE: In this study, we used the *bab1-GAL4* line which was generated by the Laski lab (Cabrera et al., 2002), not the line from the Gonzalez-Reyes lab (Bolívar et al., 2006). Consistent with the literature and demonstrated clearly in our study, the line from the Laski lab showed excellent specificity for adult TF and Cap cells.**

15-Fig 5E', F' and G': Consider black and with panels to enhance contrast

**AUTHORS’ RESPONSE: We have updated these panels with a black and white version to enhance the contrast.**

16-Lines 334-342: Please be careful. Since bab1-Gal4 is expressed (at least) in TF and cap cells, you cannot conclude that "miR-124 normally acts … in cap cells". The same comment applies to line 346.

**AUTHORS’ RESPONSE: Thank you. We have changed these places with TF and Cap cells.**

17-Lines 348-349: Revise grammar "was significantly increased its expression".

**AUTHORS’ RESPONSE: It has been corrected as suggested.**

18-Line 358: Typo in Fig 7E (germarium, not gerarium).

**AUTHORS’ RESPONSE: Thank you. It has been corrected as suggested.**

Reviewer #2: General comments

Chen et al first conducted the screening of 90 miR mutants, and among those, they identified miR-124 as an essential factor required for proper differentiation of GSCs. miR-124 is known to function for neuroblast proliferation in Drosophila as well as for tissue stem cells in other animals. Loss of miR-124 activity in Cap cells, the GSC niche, led to differentiation defect of germ-line cells, caused by upregulation of Dpp activity through notch signaling in Escort cells, the another due to unchecked epidermal growth factor receptor (EGFR) in Cap cells. This activates anti-differentiation signals in neighboring cells through Notch signaling, refining the boundary between self-renewal and differentiation niches. This manuscript describes a novel mechanism how the GSC niche regulates Dpp signaling to promote GSC differentiation. Of note, the authors found that Cap cells, the GSC niche, communicate with the neighboring escort cells (ECs), which promote differentiation of CBs and cysts (differentiation niche): the expression in DI is repressed in Cap cells by mir-124, inhibiting notch signaling activation to avoid Dpp expression ECs. The manuscript is well-written, and the logical flow is understandable. In Discussion, a diverse array of related aspects, though not the primary focus of this study, were adequately discussed. Though some of the proper controls in several genetical analyses should be conducted, this study would be a general interest to be published in PLOS Biology.

**AUTHORS’ RESPONSE: We thank the reviewer for being interested in our work and for the supportive comments to improve our work.**

Major points

1. The schematic drawing of miR-124 locus, the main focus of this study, should be shown somewhere in the figure, including information of loss-of function alleles examined in this study.

**AUTHORS’ RESPONSE: We have provided a schematic drawing of the *miR-124* locus in the revision (S18 Fig).**

2. Figure 1 and S2 clearly showed the defects in GSC maintenance and/or proliferation of the examined miR mutants. However, this study did not describe or argued about fertility. Egg numbers and the hatching rate should be described for the main focus, miR-124 mutant. And if it's not affected, it should be argued in Discussion why those defects in early differentiation does not affect fertility.

**AUTHORS’ RESPONSE: Thank you for the suggestion. We tested the fertility of *miR-124* mutants. Twenty 2-day-old *miR-124* *Drosophila* females and five 2-day-old *miR-124* males were placed into a fly bottle, which covered a dish containing fly food medium. The food dishes were replaced daily for a period of 5 days, the total number of eggs deposited on the dishes each day were counted, and the hatching larvae were counted 2 days after egg collection. From the results, we found that the egg numbers and hatching rate were significantly reduced in *miR-124* mutants in comparison to the *w*^1118^ control. We have described these results in both main text (lines 155-157) and new S2 Fig.**

3. Page 10, Lane 284, MiRNAs usually cause mRNA deadenylation and decay [67]. miRNAs could perturb efficient translation, resulting in failure of the protein expression of the target. This statement is too simplified and needs to be rephrased.

**AUTHORS’ RESPONSE: We have rephrased the statement as** “*MiRNAs usually bind to specific sequences within the 3’ UTRs of mRNAs, leading to degradation of the targeted mRNAs and reducing translation into proteins*”. **Please see lines 301-302.**

4. The authors nicely showed that luciferase reporter containing 3'UTR of fly EGFR in HEK293 was repressed in the presence of miR-124, and the repression was relieved upon substitution of the nucleotide sequence of the potential miR-124 target site. However, it does not exclusively indicate that the direct target of miR-124 is EGFR 3'UTR in fly vivo. Hence, the subheading, EGFR is a target of miR-124 in Cap cells, is currently overstatement. Preferably it needs to be examined in vivo by making transgene containing reporter and EGFR 3'UTR and its mutant in miR-124 mutant background. In addition, the rescue experiment for pERK should be conducted; the expression of miR-124 in Cap and TF, but not in EC, in miR-124 mutant background should reduce the pERK signal in EC.

**AUTHORS’ RESPONSE: We agree. We have changed the heading to** “*EGFR is a potential direct target of miR-124 in Cap cells*”, **and we also changed last sentence in this section as** “*We deduce that miR-124 normally limits EGFR pathway activity in Cap cells and likely does so by directly interacting with the EGFR 3’ UTR to reduce expression*”.

**We believe that the reviewer wanted us to do the rescue experiment for pERK in Cap cells. We have performed this additional experiment to test the pERK levels with the expression of miR-124 in Cap cells and TF in *miR-124* mutant background. The results showed that expression of miR-124 could rescue the pERK signal in Cap cells. We have mentioned these results in both main text (lines 339-341) and new Figs 4H-4J.**

5. Related with the experiment using HEK293 cells above, I wonder if overexpression of miR-124 in *Drosophila* S2 cells can repress endogenous or transfected EGFR. If this works, mutation analysis could be done in S2 cells. Because there is no report for the direct regulation of EGFR by miR-124, this is an important piece of the data in this study. In addition, is the target site of miR-124 in EGFR 3'UTR conserved? If so, it would be better to show the alignment among different species.

**AUTHORS’ RESPONSE: Thank you. We have performed the additional experiments in *Drosophila* S2 cells. We found the transcript levels of *EGFR* were significantly decreased in miR-124 and *EGFR^WT^* co-transfected cells, but not changed in miR-124 and *EGFR^MUT^* co-transfected cells. We have described these results in both main text (lines 322-325) and new S12 Fig.**

**It is interesting to test whether the target site of miR-124 in *EGFR* 3’ UTR is conserved. We then collected the *EGFR* 3’ UTR in 20 insect species represent six orders, including Diptera (13), Lepidoptera (2), Hymenoptera (2), Coleoptera (1), Hemiptera (1) and Orthopterodea (1). The alignment results showed that the target site of miR-124 in *EGFR* 3’ UTR is conserved in many *Drosophila* species, but have low or no similarity to other insects. We have described these results in both main text (lines 325-332) and new S13 Fig.**

6. What happens when EGFR is over-expressed in ECs?

**AUTHORS’ RESPONSE: We have performed the additional experiments to determine the consequences of overexpressing EGFR in ECs by using the combination of *UAS-EGFR* and *C587-GAL4*. We found that EGFR overexpression in ECs caused no effect in germline differentiation. We have described these results in both main text (lines 362-365) and new S16 Fig.**

Minor points

1. Page 6, Line 131-132, suggesting a role in the stem cell niche.: the observation alone does not suggest a role in the niche, but suggest a role for GSC maintenance and/or proliferation.

**AUTHORS’ RESPONSE: It has been corrected as suggested.**

2. Fig1B, D, Fig 3A and many others: the labels should be w1118, not W1118.

**AUTHORS’ RESPONSE: Thank you. It has been corrected as suggested.**

3. For a clearer presentation, the staining data should be presented in black and white when only one staining is shown; for example, Figure 1H', 3A'-C', 5E'-G', 7A'-D' and elsewhere applicable in Supplementary figures.

**AUTHORS’ RESPONSE: This was raised by another reviewer. We have updated these panels with a black and white version to enhance the contrast in our revised manuscript.**

4. Page 11, Lane 325-326, EGFR overexpression also significantly increased dpp RNA levels in extracts from the germarium and early egg chambers (Fig 6D).: dpp level in RNA isolated from the germarium and early egg chambers.

**AUTHORS’ RESPONSE: It has been corrected as suggested.**

5. Related to the Major Point #4, Page 11, Line 340-342, These results indicate that miR-124 normally acts directly on a defined element of the EGFR 3' UTR to limit EGFR signaling in Cap cells and thereby prevent ectopic Dpp expression in ECs and delayed germline differentiation.: This is overstatement, because the authors did not show the perturbation of the EGFR repression by mutation of miR-124 target site in 3'UTR of EGFR in vivo. A possibility of indirect regulation of EGFR by miR-124 cannot be excluded.

**AUTHORS’ RESPONSE: Thank you. We have modified the sentence to avoid over statement (also see lines 379-382 in the revised manuscript).** “*These results indicate that miR-124 normally restricts EGFR expression, likely through direct interaction with the EGFR 3’ UTR, to limit EGFR signaling in TF and Cap cells, thereby preventing ectopic Dpp expression in ECs and delayed germline differentiation.*”

Reviewer #3: In this study, Chen and colleagues identified one microRNA (miR124) expressed in the somatic niche of germline stem cells in *Drosophila*. They show that miR124 is required for the differentiation of the posterior daughter cell after the stem cell division. They further demonstrate that miR124 acts through a signaling relay mechanism, which starts by inhibiting EGFR in the anterior niche cells. The inhibition of EGFR then inhibits the synthesis of Delta, downregulating Notch activity in the posterior niche cell, which then downregulates Dpp. Ultimately downregulation of Dpp allows for the differentiation of the posterior daughter cell.

The quality of the data is of very high standards, whether they are microscopy, genetics or transcriptomic analysis. The results are convincing, well-controlled, and interpreted rigorously. It is an impressive feat given that the authors use a wide variety of techniques to decipher the underlying mechanisms. I don't have any major criticisms regarding the data and methods used as presented.

Overall, the authors' conclusions and model fit well with the current paradigm of how the GSC niche works in *Drosophila*. There is one anterior somatic niche maintaining undifferentiated GSCs and one posterior niche "allowing" differentiation rather than "inducing" differentiation. The EGF and Notch pathways were already identified as important for niche establishment and functions. However, the scope of this study could be more important if they could integrate their results with previously published data. In particular, in (Ward et al, Current Bio 2006) or (Song et al. Development, 2005), Delta has been proposed to signal from germline cells to somatic niche cells to regulate the formation and function of the niche. In my opinion, it is difficult to see how delta signaling from germline cells could fit in the authors' model on Figure 7J. It would be very important for the significance of this study to, at least, discuss it.

**AUTHORS’ RESPONSE: We appreciate the positive evaluation and the suggestions of how to improve our study. We have discussed the interesting findings from Ward et al, (Current Biology, 2006) and Song et al. (Development, 2007) in the revised manuscript (lines 484-495), including the subsequent mechanistic studies of Yatsenko et al (PLoS Genetics, 2021). We do not see any contradiction between the role of Notch signaling in specifying and maintaining Cap cells compared to the role of miR-124 in suppressing Dl signaling from Cap cells. In fact, these are potentially complementary and illustrate interesting developmental modifications of Notch signaling by microRNAs among fairly similar and juxtaposed cells.**

Other comments:

1) In figure 2, the authors use an elegant combination of bab1-Gal4 and Gal80ts, but not in figure 6, when KD or OE of EGFR. The same is true on Figure 7. Is there a specific reason?

**AUTHORS’ RESPONSE: We thank the reviewer appreciating our GAL4/GAL80^ts^ system. However, it takes a long time to do the fly recombination and crosses for such tests. We agree that it would be better to use GAL4/GAL80^ts^ system in all experiments. In this revision, we did extra experiments to use the GAL4/GAL80^ts^ system to drive *UAS-EGFR* expression. Similar results were found in comparison to the normal *GAL4* system. We have described these results in both main text (lines 354-355) and new S14 Fig.**

2) miR124 mutant are viable, but are they fertile? Is there any other defect during oogenesis?

**AUTHORS’ RESPONSE: Thank you. Another reviewer also raised the same question. We have designed the experiments to test the fertility. Twenty 2-day-old *miR-124* *Drosophila* females and five 2-day-old *miR-124* males were placed into a fly bottle, which covered a dish containing fly food medium. The food dishes were replaced daily for a continuous 5 days, the total eggs and the hatching larvae on the dishes were counted for each. From the results, we found that the egg-laying and hatching rate were significantly reduced in *miR-124* mutants in comparison to the *w^1118^* control. We have described these results in both main text (lines 155-157) and new S2 Fig.**
