# Supplementary material for: Communication between the stem cell niche and an adjacent differentiation niche through miRNA and EGFR signaling orchestrates exit from the stem cell state in the Drosophila ovary
Source: PLoS Biol. 2024 Mar 21;22(3):e3002515. doi: 10.1371/journal.pbio.3002515 (PMC10986965; doi:10.1371/journal.pbio.3002515)
Supplement: Attachment — Submitted filename: Response to Reviewers.docx [file pbio.3002515.s026.docx]

Reviewer #1: I appreciate the care and thoughtfulness of the AUs in their reply to my comments. I am happy with the current version of the MS, which has came out as a round and very interesting story. Congratulations.

**AUTHORS’ RESPONSE: We thank the reviewer for a series of constructive comments that helped to improve our manuscript.**

Reviewer #2: The authors have satisfactorily addressed most of my concerns, though the in vivo analysis for EGFR 3'UTR has not been addressed. However, the authors examined that point in S2 cells and investigated the conservation of 3'UTR. In addition, other genetical analyses strongly support their claim. I personally think the revised manuscript is, in principal, now ready for publication.

**AUTHORS’ RESPONSE: We thank the reviewer for a series of constructive comments that helped to improve our manuscript.**

Reviewer #3: In this revised manuscript, the authors have addressed my main concerns experimentally and successfully. However, in contrast to the authors, I am still puzzled by how Escort Cells can integrate Delta signals from Cap cells AND Germline cells at the same time? It is not shown in their final model (Figure 7J). It is nonetheless a minor point in this impressive piece of work.

**AUTHORS’ RESPONSE: We thank the reviewer for a series of constructive comments that helped to improve our manuscript. Regarding Delta signals to ECs, there is doubtless more to be discovered, but we are not aware of any currently known contradictions. Just prior to pupation, a complex series of interactions have been shown first to specify TF cells, involving germline signals that activate Notch, and then for the most posterior TF to produce Notch ligand and specify Cap cells from EC/Cap cell progenitors (Zamfirescu et al., 2022; Yatsenko & Shcherbata, 2018). All of these events are important during development to organize TF, Cap and Escort cells. There are also studies showing that Notch signaling is required to maintain Cap cells and derives from GSCs (Song et al., 2007; Ward et al., 2006), with one report showing that excess Delta signals from Germline cells could produce more Cap cells during adulthood (Ward et al., 2006). From these studies, it appears that germline cells provide weak activation of Notch signaling in ECs.** **Our results reveal a mechanism for ensuring that there is no significant Delta-Notch signaling to ECs from Cap cells, consistent with the apparent objective of maintaining only low levels of Notch activation in ECs. We have adjusted the relevant paragraph in the Discussion to clarify our understanding of the status of ligand presentation for Notch activation in adult ECs (lines 477-486). Thank you.**

Zamfirescu AM, Yatsenko AS, Shcherbata HR. Notch signaling sculpts the stem cell niche. Front Cell Dev Biol. 2022;10:1027222.

Yatsenko AS, Shcherbata HR. Stereotypical architecture of the stem cell niche is spatiotemporally established by miR-125-dependent coordination of Notch and steroid signaling. Development. 2018;145:dev159178.

Song X, Call GB, Kirilly D, Xie T. Notch signaling controls germline stem cell niche formation in the *Drosophila* ovary. Development. 2007;134:1071-80.

Ward EJ, Shcherbata HR, Reynolds SH, Fischer KA, Hatfield SD, Ruohola-Baker H. Stem cells signal to the niche through the Notch pathway in the *Drosophila* ovary. Curr Biol. 2006;16:2352-8.
